# Genetically Determined Loss-of-Function of the Organic Cation Transporter OCT1 Is Associated with Lower Liver Fat Content in Humans

**DOI:** 10.3390/ijms27167422

**Published:** 2026-08-19

**Authors:** Anna K. Scheurer-Böhme, Eileen Moritz, Stefan Weiss, Robin Bülow, Marie-Luise Kromrey, M. Kamal Nasr, Uwe Völker, Henry Völzke, Stefan Engeli, Jens-Peter Kühn, Alexander Teumer, Mladen V. Tzvetkov

**Affiliations:** 1 Department of General Pharmacology, Institute of Pharmacology, Center of Drug Absorption and Transport (C DAT), University Medicine Greifswald, 17475 Greifswald, Mecklenburg-Western Pomerania, Germany; annaklara.boehme@med.uni-greifswald.de (A.K.S.-B.);; 2 Interfaculty Institute of Genetics and Functional Genomics, University Medicine Greifswald, 17475 Greifswald, Mecklenburg-Western Pomerania, Germany; 3 DZHK (German Centre for Cardiovascular Research), Partner Site North, 17475 Greifswald, Mecklenburg-Western Pomerania, Germany; 4 Institute of Diagnostic Radiology and Neuroradiology, University Medicine Greifswald, 17475 Greifswald, Mecklenburg-Western Pomerania, Germany; 5 Department of Psychiatry and Psychotherapy, University Medicine Greifswald, 17475 Greifswald, Mecklenburg-Western Pomerania, Germany; 6 Institute for Community Medicine, University Medicine Greifswald, 17475 Greifswald, Mecklenburg-Western Pomerania, Germany; 7 Department of Clinical Pharmacology, Institute of Pharmacology, Center of Drug Absorption and Transport (C DAT), University Medicine Greifswald, 17475 Greifswald, Mecklenburg-Western Pomerania, Germany; 8 Institute and Policlinic for Diagnostic and Interventional Radiology, University Hospital, Carl Gustav Carus University, TU Dresden, 01304 Dresden, Saxony, Germany

**Keywords:** metabolic dysfunction-associated steatotic liver disease, non-alcoholic fatty liver disease, hepatic steatosis, OCT1, organic cation transporter, Study of Health in Pomerania, UK Biobank, metabolic phenotype, mitochondrial respiration

## Abstract

Fatty liver is associated with increased all-cause mortality. Organic cation transporter 1 (OCT1) is a polyspecific hepatic uptake transporter. OCT1 mediates hepatic uptake of vitamin B1 (thiamine); thus, OCT1 deficiency may impair thiamine availability and consequently reduce glucose-derived energy. Consequently, OCT1-knockout mice exhibit significantly reduced liver fat. In 2% of Europeans and White Americans, common genetic variants markedly reduce OCT1 function. In this study, we used these naturally occurring variants to investigate the influences of reduced OCT1 function on liver fat in humans. We analyzed 2512 whole-body MRI datasets from the Study of Health in Pomerania (SHIP) and validated the findings using 31,594 datasets from the UK Biobank. In both cohorts, OCT1 deficiency was associated with lower fat content in the liver (*p* = 0.027 in SHIP; *p* = 2.9 × 10^−6^ in the UK Biobank), but not in other body compartments. OCT1 deficiency remained an independent predictor after adjustment for age, BMI, daily alcohol consumption, and biological sex. To investigate underlying mechanisms, we used Seahorse assays to show that OCT1-overexpressing cells exhibited increased mitochondrial metabolic activity when glucose was the sole energy source. Our findings warrant further investigation of OCT1 as a potential contributor to the pathogenesis of fatty liver.

## 1. Introduction

Fatty liver, particularly when accompanied by metabolic dysregulation—referred to as metabolic dysfunction-associated steatotic liver disease (MASLD)—is becoming an increasingly important global health concern. Its prevalence was estimated at 38% worldwide in 2023 and is projected to rise further in the coming years [1,2,3]. MASLD increases patient’s over-all mortality by a 1.9-fold [4], as well as the risk of hepatocellular carcinoma by a 3.51- and that of hepatic cirrhosis by a 4.7-fold [5]. Additionally, extrahepatic complications include cardiovascular events [6,7], type-2 diabetes mellitus [8], chronic kidney disease [9], and extrahepatic carcinomas such as gastrointestinal, skin, breast and male genital cancer [10,11].

Pharmacological therapies specifically targeting fatty liver and MASLD are urgently needed. Recently, resmetirom, a selective thyroid hormone receptor-β (THRβ) agonist, was approved for the treatment of metabolic dysfunction-associated steatohepatitis with moderate to advanced liver fibrosis [12]. Several other phase II and III clinical trials are currently in progress with therapeutic targets aiming at fibroblast growth factor 21 (FGF21) [13,14], farnesoid X receptor (FXR) [15], and chemokine receptor type 2/type 5 (CCR2/CCR5) [16]. Most of these treatments also aim to improve advanced stages of MASLD or a consequential condition such as fibrosis. There is still a need for mechanistic insights into the development of MASLD and early hepatic steatosis in order to treat the initial stages of the disease or even to prevent disease in high-risk patients.

One possible target may be organic cation transporter 1 (OCT1), a polyspecific uptake transporter in human hepatocytes. The livers of OCT1-knockout mice showed markedly reduced liver weight and triglyceride content compared with wild-type mice. This difference was even more pronounced in a leptin-deficient obese mouse model [17,18]. In humans, OCT1 is strongly and almost exclusively expressed at the sinusoidal membrane of hepatocytes [19,20]. A possible explanation for this observation in OCT1-knockout mice is the ability of OCT1 to transport thiamine (vitamin B1) [17]. Reduced OCT1 activity could decrease intracellular thiamine availability, thereby impairing thiamine-dependent metabolic pathways, including the coupling of glycolysis to the citric acid cycle. This, in turn, could alter hepatic energy metabolism and caloric balance.

Human OCT1 is highly genetically variable. There are several common genetic variants that cause a complete or substrate-specific loss of OCT1 function with effects on the transport activity of OCT1 [21,22,23,24,25,26,27,28]. The Met420del variant (known as the OCT1*2 allele) is of special interest, as it is the most common variant and results in a substrate-specific loss of OCT1 function. Based on the OCT1 genotype, individuals can be defined as poor OCT1 transporters with zero fully active OCT1 alleles, intermediate OCT1 transporters with one, and extensive OCT1 transporters with two fully active OCT1 alleles. The distribution of OCT1 genotypes differs worldwide. In Europe, 2% of the population are poor OCT1 transporters [28].

In this study, we hypothesize that humans with low OCT1 activity have reduced liver fat content. We compared liver fat content, quantified by proton density fat fraction (PDFF) of magnetic resonance imaging (MRI), in poor OCT1 transporters with that in individuals carrying one or two active OCT1 alleles, expecting higher liver fat content with an increasing genetically predicted OCT1 activity. We analyzed 2512 individuals of the Study of Health in Pomerania (SHIP) cohort [29] and confirmed our findings in a second cohort containing data from 31,594 individuals of the UK Biobank, a population-based project based in the United Kingdom [30]. Additional to testing our main hypothesis, we also performed exploratory analyses of subgroup specific effects. To explore the underlying mechanisms, we analyzed in vitro changes in mitochondrial respiration in OCT1-overexpressing cells compared with control cells.

## 2. Results

### 2.1. Characterization of the Study Populations

**Figure 1:**
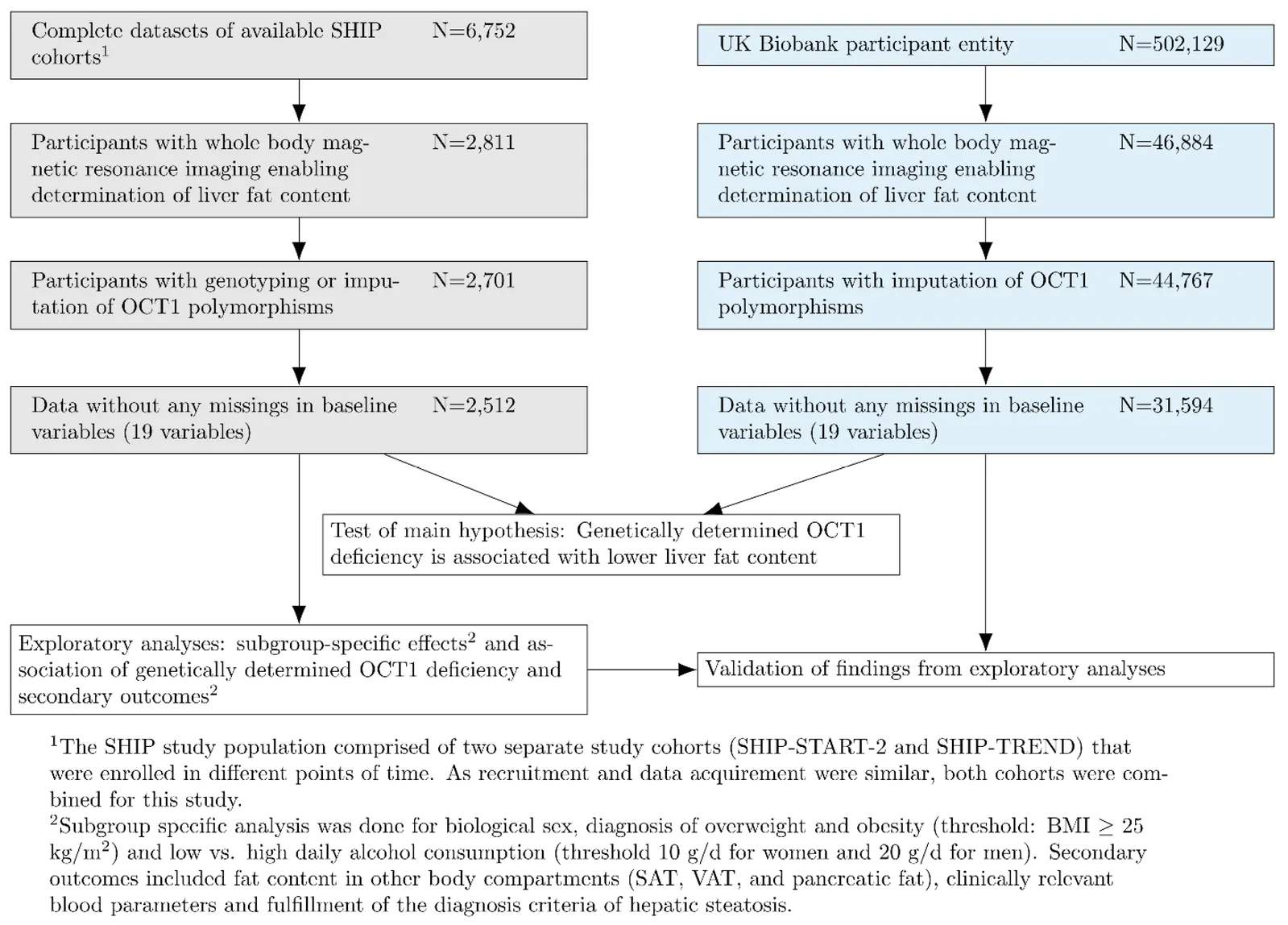
Characteristics of the study populations and workflow of statistical analysis.

Characteristics of the study populations and workflow of the statistical analysis are shown in Figure 1. We analyzed 2512 participants from the population-based SHIP. This represents 37.2% of the total number of participants (6752 participants). A total of 3941 participants (60.0%) could not be included in the analysis because of missing MRI-derived liver fat content data, another 110 (1.6%) because of missing OCT1 genotype, and 189 (2.8%) because of missing baseline variables. We analyzed 31,594 participants from the UK Biobank. This represents 6.3% of the total study population (502,129 participants) and 67.4% of those for whom MRI data were available (46,884). A further 2117 (0.4%) were excluded because of missing OCT1 data and another 13,173 (2.6%) because of missing baseline variables.

**Figure 2:**
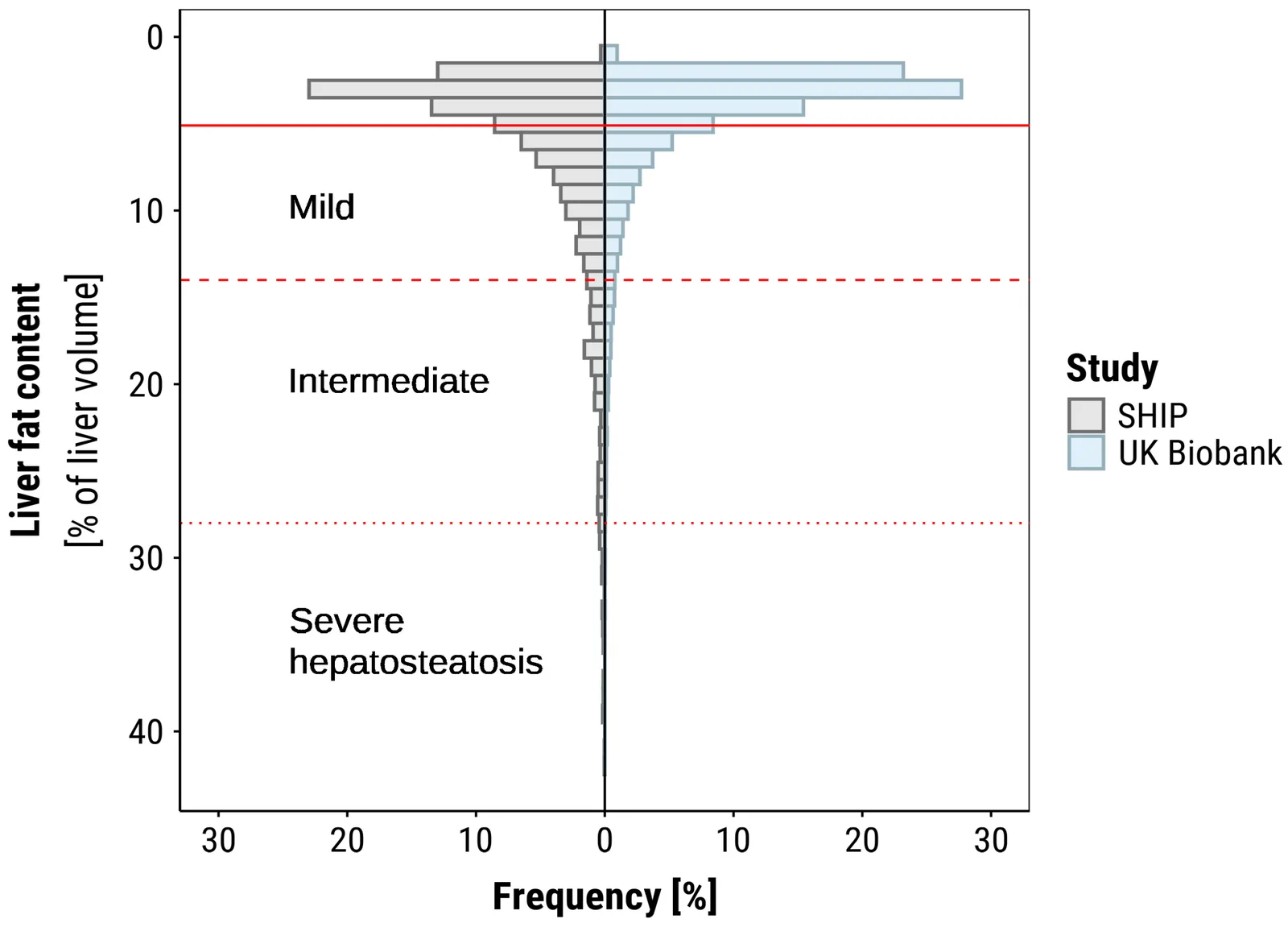
**Distribution of liver fat content in both populations included in this study.** Liver fat content was assessed by MRI. Horizontal red lines indicate diagnostic thresholds for MRI-defined hepatic steatosis (solid: mild, >5.1%; dashed: moderate, >14.0%; dotted: severe, >28.0% liver fat content). Data from the SHIP cohort are shown in gray and those from the UK Biobank in blue.

The median age of the SHIP individuals was 52 years (Table 1). Exactly 1279 study participants (51%) were women, 1017 (40.5%) participants met the criteria for mild to severe MRI-defined hepatic steatosis, defined as 5.1% of liver volume for mild, 14.0% for intermediate, and 28.0% for severe hepatic steatosis (Figure 2). Out of the participants with mild to severe MRI-defined hepatic steatosis 407 (40.0%) were women.

In comparison to the SHIP study, UK Biobank participants were older, with a median age of 66 years. A total of 16,104 study participants (51%) were women. Only 7502 (23.7%) participants fulfilled the criteria for MRI-defined hepatic steatosis, which is a notably smaller percentage than in SHIP. Of these, 40.9% were women. Study participants of the UK Biobank had lower median body mass index (BMI, 25.9 kg/m^2^ vs. 27.2 kg/m^2^) and fatty liver index (39.4 vs. 47.1) than those of the SHIP study. The median self-reported alcohol consumption was higher in the UK Biobank population (11.0 vs. 4.3 g/d). The differences between women and men were similar in both study cohorts, with women having lower BMI, fatty liver index (FLI) and daily alcohol consumption.

### 2.2. Genetically Predicted OCT1 Deficiency Was Associated with Lower Liver Fat Content

The main hypothesis of our study was that individuals with genetically determined OCT1 deficiency would have significantly lower liver fat content than those with full OCT1 activity. Consequently, we compared MRI-quantified liver fat content across groups stratified by genetically predicted OCT1 activity score. Among the 2512 participants in SHIP, 72.7% carried two, 25.0% carried one and 2.2% carried zero fully active OCT1 alleles; among the 31,594 participants of the UK Biobank, 76.7% carried two, 21.7% one and 1.6% zero fully active OCT1 alleles (*1 and *2 genotypes were defined as fully active, see Supplementary Table S5 for detailed OCT1 genotype distribution in both study populations).

**Figure 3:**
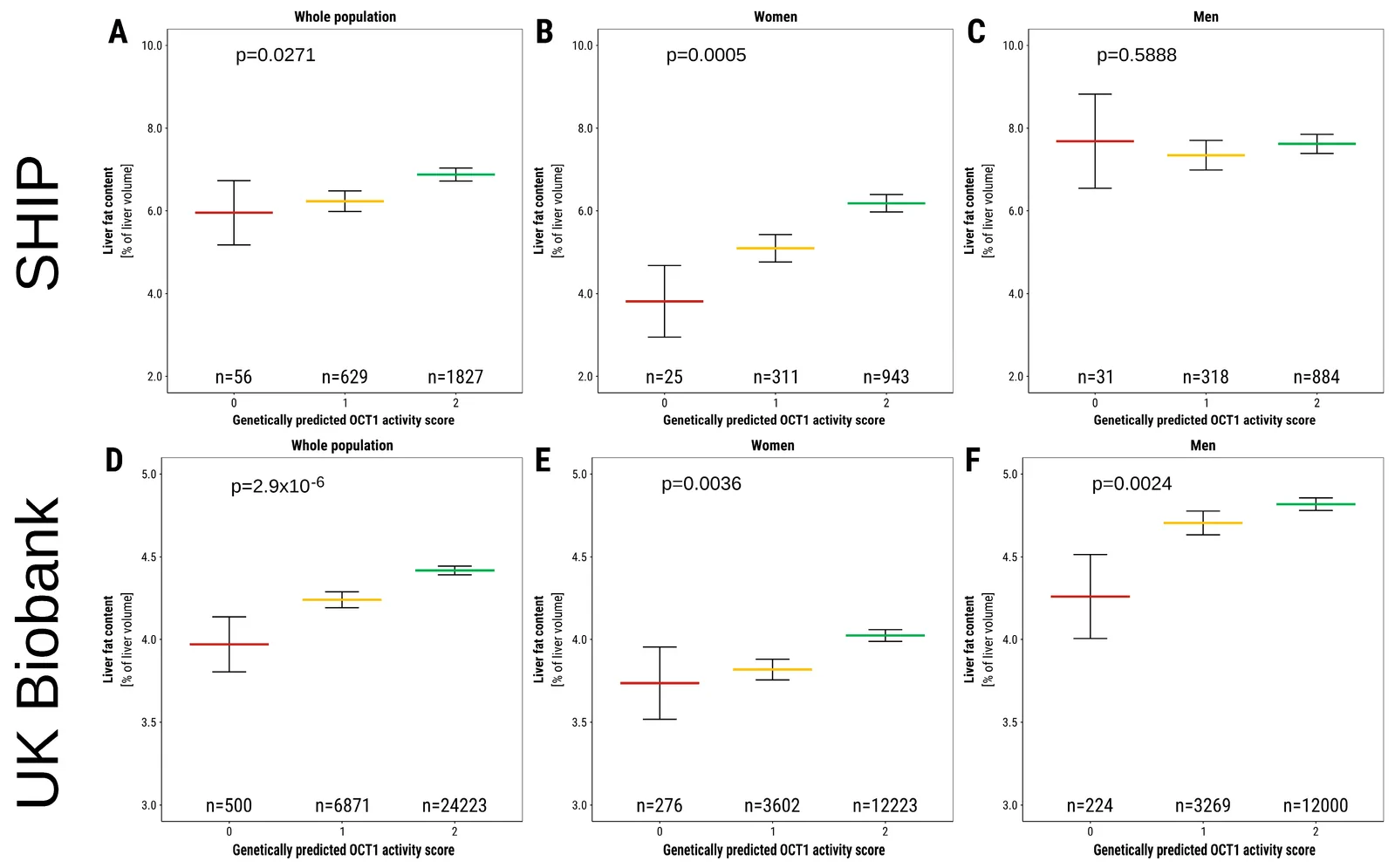
**Association between liver fat content measured by MRI and genetically predicted OCT1 activity score.** Analyses were performed in the overall study population (**A**,**D**) and stratified by sex (women: (**B**,**E**); men: (**C**,**F**)). Associations were first evaluated in SHIP (**A**–**C**) and subsequently validated in the UK Biobank (**D**–**F**). Liver fat content for individuals with genetically predicted activity scores of zero, one, and two fully active OCT1 alleles are indicated in red, yellow, and green, respectively. Data are presented as mean ± SEM. *p* values were calculated using the Jonckheere–Terpstra trend test.

In SHIP, relative liver fat content was significantly lower in groups with fewer fully active OCT1 alleles (Figure 3A–C, Table 2). This result was replicated in UK Biobank participants. The effect size was r = 0.038 in SHIP and r = 0.026 in the UK Biobank (Figure 3D,E, Table 2).

### 2.3. Multiple Regression Revealed Genetically Predicted OCT1 Activity as an Independent Predictor of Hepatic Steatosis

Additionally, we tested whether our findings were robust after adjustment for confounders previously known to influence liver fat content in humans. We therefore performed multiple logistic and linear regression analyses that included the following covariates known to affect fatty liver disease: age, BMI, daily alcohol consumption and sex [31]. In the linear regression analysis, OCT1 was an independent predictor of liver fat content both in SHIP and UK Biobank (Table 3). The increase in explanatory power of the model (ΔR^2^) after adding genetically predicted OCT1 activity score to the model was relatively low (0.00192 for SHIP and 0.00059 for the UK Biobank, respectively). However, it is of note that the overall explained variance of the models (R^2^) was also limited (0.3651 and 0.27982 for SHIP and UK Biobank respectively).

Higher genetically predicted OCT1 activity score was a predictor of the diagnosis of MRI-defined hepatic steatosis in both study populations. The risk of MRI-defined steatosis increased with each increment in activity score with an odds ratio (OR) of 1.22 (95% CI [1.01; 1.48], *p* = 0.043) in SHIP and 1.13 (95% CI [1.06; 1.21], *p* = 1.08 × 10^−4^) in the UK Biobank (Supplementary Table S7).

### 2.4. Sex-Dependent Effects on the Association Between Geneticially Predicted OCT1 Deficiency and Liver Fat Content

Next, we explored sex-dependent effects by including sex as a stratification factor in our analysis. Without further adjustments, the association of genetically predicted OCT1 activity score and liver fat content was sex-dependent in the SHIP study (Table 4, Figure 3). Women who had an activity score of 0 had a 32% lower median liver fat content compared to those with an activity score of 2. In contrast, no significant association between liver fat content and genetically predicted OCT1 activity score could be found for men. Our multiple linear regression, which controlled for confounding variables, showed similar findings (Supplementary Tables S6 and S7). In the SHIP study, genetically predicted OCT1 activity score was an independent predictor of liver fat content for the women but not for the men (β = 0.072, *p* = 0.001 and β = 0.021, *p* = 0.354, respectively).

In the UK Biobank cohort, we could not confirm the strong sex dependence. In this cohort, both women and men showed a significant association between genetically predicted OCT1 activity score and liver fat content. However, the increase in explanatory power in the linear model was also greater in women than in men (ΔR^2^ = 0.00070 for women and ΔR^2^ = 0.00051 for men, *p* < 0.001 for all models).

### 2.5. Effects of the OCT1 *2 Allele on the Association with Liver Fat Content

**Figure 4:**
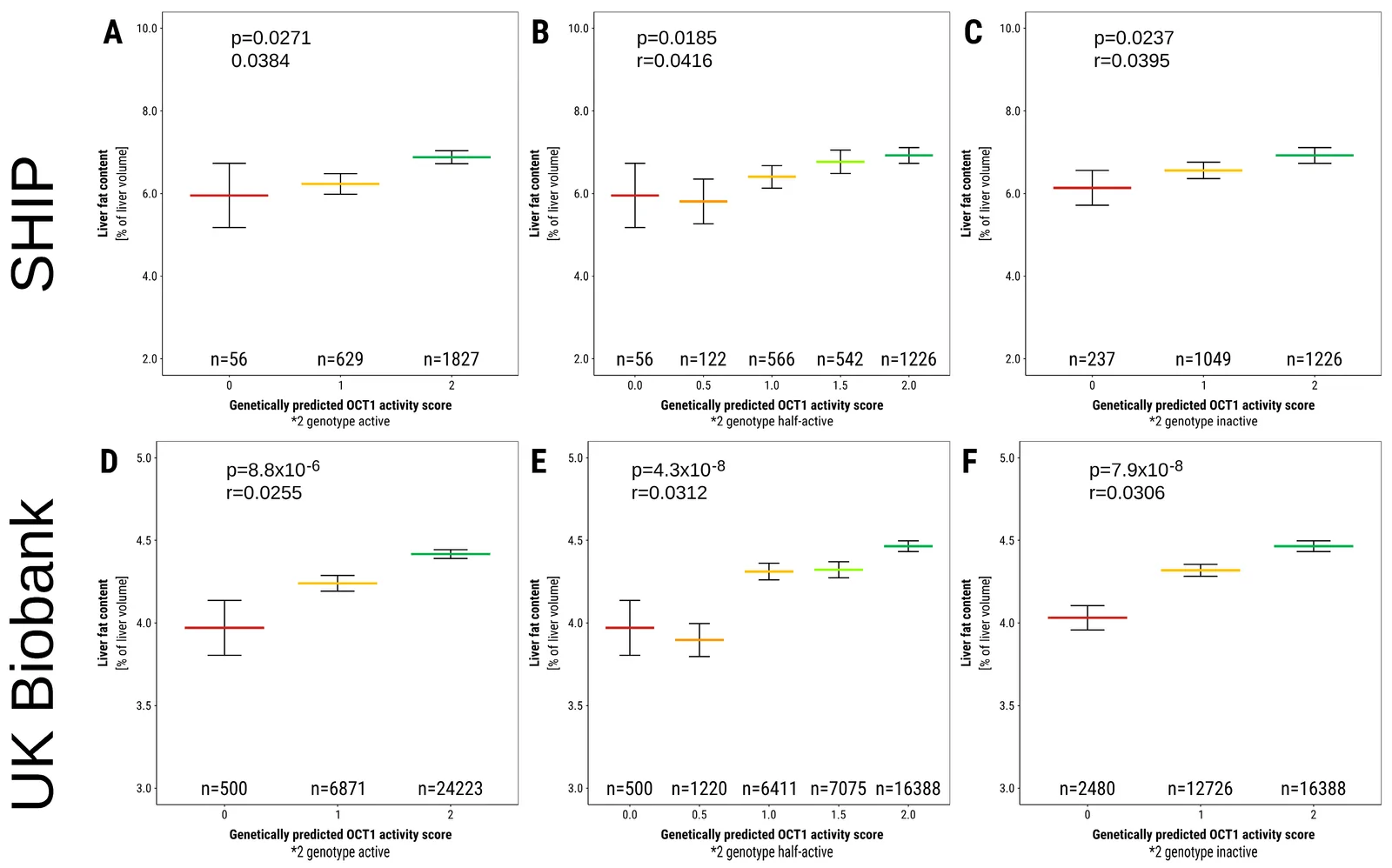
Influence of the *2 allele on the association between OCT1 deficiency and liver fat content measured by MRI. Liver fat content was analyzed in relation to the genetically predicted OCT1 activity score, with the *2 genotype modeled as fully active (**A**,**D**), partially active (**B**,**E**), or inactive (**C**,**F**) in SHIP (**A**–**C**) and the UK Biobank (**D**–**F**). Data are presented as mean ± SEM. *p* values and effect sizes for the association with OCT1 activity were calculated using the Jonckheere–Terpstra trend test.

The OCT1*2 allele has substrate-specific effects on OCT1 activity. Without knowledge of the OCT1 substrate that mechanistically accounts for the observed differences in liver fat content, it is not possible to functionally assess OCT1*2 activity in vitro. To evaluate the role of the *2 allele in the association between OCT1 and liver fat content, we performed three independent analyses in which we considered the OCT1*2 allele as fully active (genetically predicted OCT1 activity score of 1), half-active (genetically predicted OCT1 activity score of 0.5), or completely inactive (genetically predicted OCT1 activity score of 0) in both study populations (Figure 4). The effects of genetically predicted OCT1 activity score on liver fat content were observed in all three models in both studies. In the SHIP study, the effect size was the highest when considering the *2 genotype as half-active (Figure 4A, r = 0.0416, *p* = 0.0185), followed by *2 as inactive (Figure 4B, r = 0.0395, *p* = 0.0237) and *2 as active (Figure 4C, r = 0.0384, *p* = 0.0271). However, the variation in association strength across the three models was relatively small. In the UK Biobank, similar trends were observed and the effect size was highest when considering *2 as half-active (Figure 4D–F).

### 2.6. Effects of Genetically Predicted OCT1 Activity Score in Relation to Daily Alcohol Consumption

We further explored in detail the role of daily alcohol consumption. Liver fat content was analyzed in relation to genetically predicted OCT1 activity score after stratification by low and high daily alcohol consumption (thresholds of >20g/d for men and >10g/d for women). The association between genetically predicted OCT1 activity score and liver fat content was only present in participants with low daily alcohol consumption in the SHIP study, while no significant association could be found in participants with high daily alcohol consumption (Table 4). Additionally, a strong sex-dependency could be found in the subgroup of participants with low daily alcohol consumption, as only women showed an association between genetically predicted OCT1 activity score and liver fat content (r = 0.100, *p* = 0.0004 for women and r = −0.004, *p* = 0.5491 for men, respectively).

We could not confirm a dependence on daily alcohol consumption in the UK Biobank cohort, as both subgroups of low and high consumption showed a significant association between genetically predicted OCT1 activity score and liver fat content (Table 4). However, we could confirm the sex-dependency in the subgroup of participants with low alcohol consumption. After additional stratification for sex, an association between genetically predicted OCT1 activity score and liver fat content could only be found in women (r = 0.022, *p* = 0.0162) and not in men (r = 0.011, *p* = 0.1446, Table 4).

### 2.7. Effects of Genetically Predicted OCT1 Activity Score in Relation to Body Weight

In assessing the role of overweight, stratification of non-overweight and overweight individuals (threshold BMI ≥ 25 kg/m^2^) revealed no clear subgroup-specific effect of genetically predicted OCT1 activity on liver fat content (Table 4). However, after additional stratification for sex, a strong association between genetically predicted OCT1 activity score and liver fat content could be detected in overweight women in both study cohorts. In comparison with all subgroup analysis performed in the present study, overweight women were the subgroup with the largest effect size of the reported association in both cohorts analyzed (r = 0.110, *p* = 0.0011 for SHIP study; r = 0.042, *p* = 5.48 × 10^−5^ for UK Biobank, Table 4).

### 2.8. Lack of Effect of Genetically Predicted OCT1 Activity Score on Other Fat Compartments and Parameters of Metabolic Dysfunction

We analyzed the influence of genetically predicted OCT1 activity on fat content in other body compartments—including subcutaneous adipose tissue, visceral adipose tissue, and pancreatic fat content. No significant association between genetically predicted OCT1 activity score and any of these fat compartments was observed in either SHIP or UK Biobank. There were also no significant effects after stratification by sex (Supplementary Table S9). We also analyzed parameters of metabolic dysfunction. Only plasma triglycerides showed an association with genetically predicted OCT1 activity score in both study cohorts (Supplementary Table S8).

### 2.9. OCT1-Overexpressing HEK293 Cells Had Increased Mitochondria-Based Metabolic Activity When Glucose Was the Only Available Energy Source

**Figure 5:**
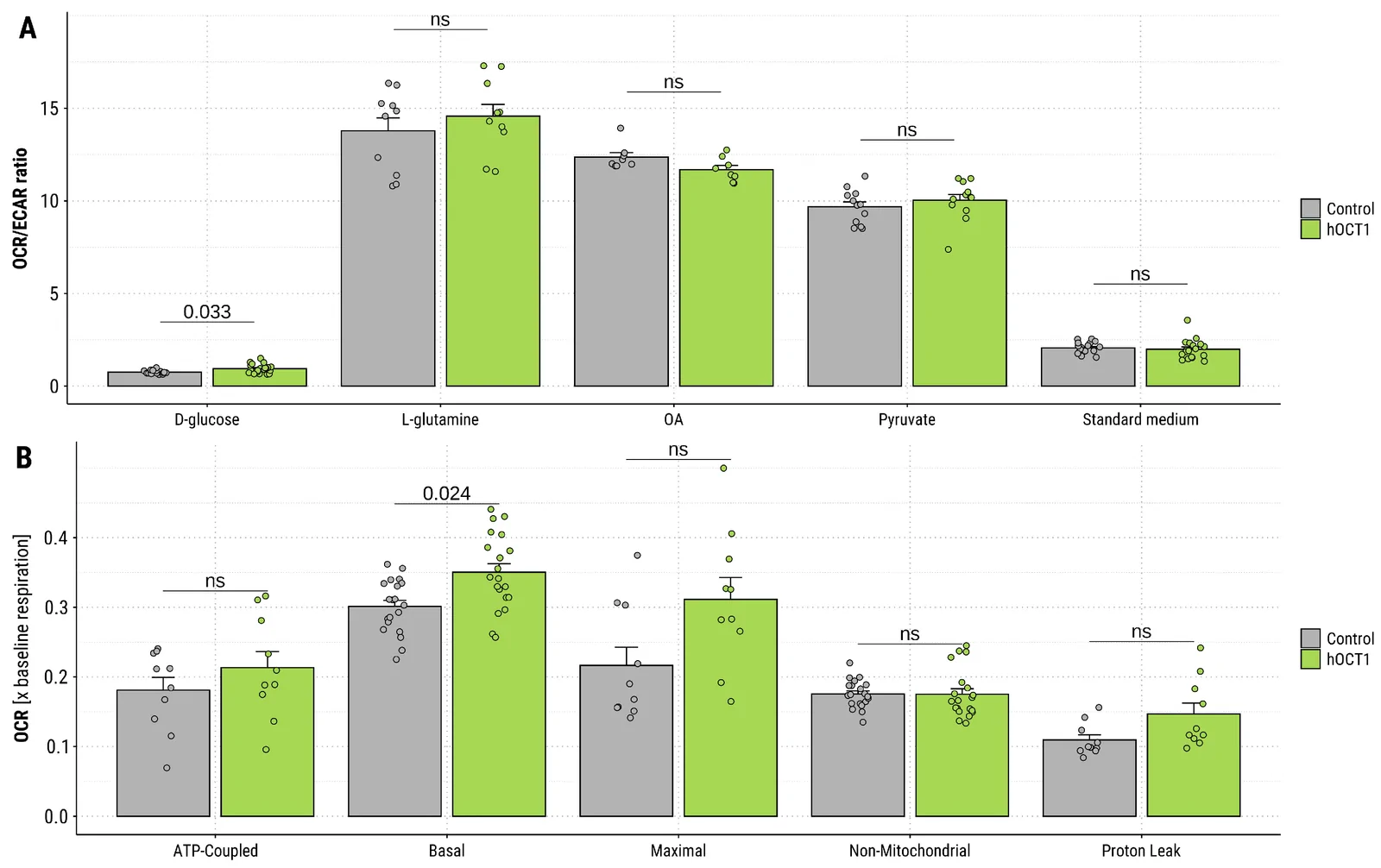
**OCT1 overexpression leads to increased mitochondrial-based metabolic activity when glucose is used as a sole energy source.** A previously established Agilent Seahorse protocol was applied to HEK293 cells stably overexpressing human OCT1 (green bars) and control cells (pcDNA5, gray bars) to assess the OCR/ECAR ratio (**A**) and key parameters of mitochondrial respiration (**B**). (**A**) Following a 1 h starvation period, cells were incubated with a single energy source. The basal metabolic phenotype was determined as the ratio of mean oxygen consumption rate (OCR) to mean extracellular acidification rate (ECAR), calculated from five measurement cycles prior to the addition of respiratory chain inhibitors. Higher OCR/ECAR ratios indicate a more oxidative, mitochondria-dependent phenotype; whereas, lower ratios reflect a greater reliance on glycolysis. (**B**) Mitochondrial respiration parameters under glucose conditions. Following a 1 h starvation period, cells were incubated with glucose as the sole energy source. Consequently, inhibitors of the respiratory chain were injected to assess the OCR coupled to specific mitochondrial functions. Data are presented as mean ± SEM from 3 to 6 independent experiments performed in duplicate. *p* values were calculated using unpaired Welch’s *t*-tests with Bonferroni correction for multiple comparisons. ns, *p* ≥ 0.05.

The impact of human OCT1 (hOCT1) on energy production in HEK293 cells was analyzed using the developed protocol for Agilent Seahorse. The metabolic phenotype of the cells was assessed with different available energy sources (D-glucose, L-glutamine, oleic acid [OA], pyruvate, and standard assay medium). The ratio of oxygen consumption rate (OCR) to extracellular acidification rate (ECAR) was analyzed to assess the basic metabolic phenotype of the cells. Higher ratios point to a more mitochondrial-based metabolic activity, while lower values indicate greater reliance on glycolytic energy production. The OCR/ECAR ratio was significantly higher in hOCT1-overexpressing cells compared to control cells when D-glucose was the only available energy source (Figure 5A, mean ± SEM: 0.94 ± 0.05 vs. 0.76 ± 0.02 for hOCT1 vs. control, unpaired Welch’s *t*-test, adjusted *p* = 0.033). The OCR/ECAR ratio did not differ significantly for the other energy sources and standard DMEM.

The higher OCR/ECAR ratio in hOCT1-overexpressing cells when utilizing D-glucose could be explained by a higher mitochondrial activity. hOCT1-overexpressing cells showed a significantly higher basal respiration than control cells (Figure 5B, mean ± SEM: 0.35 ± 0.01 vs. 0.30 ± 0.01 for hOCT1-overexpressing and control cells, respectively, unpaired Welch’s *t*-test, Bonferroni correction for multiple testing [n = 10], adjusted *p* = 0.024). No significant differences could be found for any other analyzed parameter. However, there was a trend towards lower glycolytic ATP production rates and higher ATP-coupled and maximal respiration in hOCT1-overexpressing HEK293 cells (Supplementary Figure S1). Accordingly, no significant differences could be found in the analyzed parameters of ATP production rate and mitochondrial respiration with different sole energy sources (Supplementary Table S10).

## 3. Discussion

We report that common genetic polymorphisms leading to OCT1 deficiency are reproducibly associated with lower liver fat content in humans. This association was observed in two independent population cohorts comprising more than 34,000 individuals, including 556 with genetically determined OCT1 deficiency. The association was independent of age, BMI, and daily alcohol consumption.

Genetically predicted OCT1 deficiency was selectively associated with lower liver fat content in our study, without affecting subcutaneous, visceral, or pancreatic fat (Supplementary Table S9). This finding is fully consistent with the nearly exclusive expression of OCT1 in the liver [19,20]. The highly liver-specific expression pattern of OCT1 and the absence of notable pathological consequences in individuals with OCT1 deficiency provide a rationale for further investigation into the role of OCT1 in the pathogenesis of MASLD and its potential as a therapeutic target.

Previous studies have shown reduced liver fat content in OCT1-knockout mice [17,18], suggesting a causal role of OCT1 deficiency in regulating hepatic lipid accumulation. In our study, leveraging the unusually common genetic variability of OCT1 in humans, we demonstrate that this effect translates to the human population. However, the effect size of OCT1 deficiency in humans appears to be smaller than that reported in mice. The increase in explanatory power (ΔR^2^) after including OCT1 in the linear regression models was modest (0.00192 in SHIP and 0.00059 in the UK Biobank). In contrast, in the OCT1-knockout mice, a reduction of 25% in liver-to-body weight ratio was observed, which can be attributed to changes in liver fat content [17]. First, not all human OCT1 polymorphisms included in our analyses lead to complete loss of function [28]. It could be speculated that full pharmacological inhibition of OCT1 in humans may exert a stronger effect on liver fat content than the partial genetic deficiency observed here. OCT1 inhibition has also been suggested as an additional mechanism by which the commonly used antidiabetic drug metformin may act in the liver [32]. However, metformin is a very low-potency inhibitor of OCT1, and more potent inhibitors would be required. Second, substrate specificity differs substantially between human and mouse OCT1 [33,34], and organ expression patterns also diverge. While OCT1 is almost exclusively expressed in the human liver [19,20], mice also show expression of OCT1 in the proximal tubules of the kidney [35,36]. Third, a non-selected human population cohort inherently exhibits much greater variability in complex traits such as liver fat content compared with genetically defined mouse models kept under standardized environmental conditions [37]. This broader distribution is clearly visible in both cohorts analyzed (Figure 2; Table 1).

The size of the OCT1 effect on liver fat is comparable to that of other well-characterized genetic determinants of MASLD. Genome-wide association studies have identified several loci associated with MASLD risk, such as PNPLA3, TM6SF2, APOE, GPAM, FTO and HSD17B13 [38,39,40], with PNPLA3 and TM6SF2 showing the strongest effects [41,42]. Among them, PNPLA3 has already been discussed as a promising drug target for MASLD treatment [43]. The association of the genetically predicted OCT1 activity score with liver fat content quantified by MRI observed in our study (Beta = 0.044 for SHIP and 0.024 in the UK Biobank) is lower than the effect sizes reported for PNPLA3 (Beta = 0.125) and TM6SF2 (Beta = 0.106), but comparable to APOE (Beta = −0.039) and GPAM (Beta = 0.038), as well as higher than the effect of FTO (Beta = 0.013) [44]. For HSD17B13, no association with liver fat content had been detected previously, which is most likely due to its major contribution to cirrhosis rather than lipid accumulation of the liver [40].

It should also be considered that relatively small increases in liver fat can already lead to the development of MASLD. A hepatic lipid content of only 5.1% is considered pathological [45]. Accordingly, a low genetically predicted OCT1 activity score acted protectively with respect to the diagnosis of MRI-defined hepatic steatosis disease based on MRI-measured liver fat content in our study. The odds of meeting the criteria for MRI-defined hepatic steatosis decreased substantially—by up to 22% per genetically predicted OCT1 activity score (Supplementary Table S7). Thus, our findings identify OCT1 as a previously unrecognized genetic locus contributing to MASLD risk. Additionally, evaluation of targeted OCT1 inhibition may contribute to the portfolio of therapeutic strategies for patients with MASLD.

The organ specificity of OCT1 expression, together with the findings from OCT1-knockout mice and the confirmation of our results in two independent human cohorts, supports a causal role of OCT1 in hepatic fat accumulation. However, the biochemical mechanisms underlying this association remain unclear. OCT1 is best known as a transporter of therapeutic drugs and xenobiotics, but it also transports thiamine (vitamin B1). Reduced intracellular thiamine concentrations have previously been proposed to explain the reduced hepatic lipid accumulation in OCT1-knockout mice [17]. Thiamine serves as an essential cofactor for pyruvate dehydrogenase, which links glycolysis to the tricarboxylic acid cycle and, through the acetyl-CoA pool, to mitochondrial oxidative phosphorylation. Because lipolysis and lipogenesis are less thiamine-dependent, OCT1-related thiamine depletion may drive a compensatory increase in lipolysis or, more likely, a decrease in lipogenesis, offering a possible mechanistic explanation for the reduced liver fat content observed in OCT1-deficient individuals in our study. This model—in which OCT1-dependent, thiamine-mediated coupling links glycolysis to oxidative phosphorylation—is supported by our in vitro data: OCT1-overexpressing HEK293 cells showed higher mitochondrial respiration when glucose served as the sole energy source; whereas, utilization of oleic acid (a readout for β-oxidation), which is less thiamine-dependent, was not significantly affected by OCT1 overexpression in this cell model (Supplementary Table S10). Further experiments directly comparing glucose alone with glucose combined with oleic acid, ideally in primary hepatocytes, will be required to substantiate these thiamine-dependent compensatory effects on lipolysis and lipogenesis and to develop a consistent mechanistic model linking OCT1 activity to hepatic energy metabolism. One apparent discrepancy is that, in contrast to OCT1-knockout mice, plasma thiamine concentrations in humans do not appear to depend on OCT1 genotype [46]. This is not necessarily inconsistent with our model, since OCT1 mediates hepatic thiamine uptake and the proposed effect operates at the intracellular level, which plasma concentrations may not reflect.

An endogenous biomarker that may reflect OCT1-dependent alterations in hepatic metabolism is isobutyryl-carnitine. Plasma levels of isobutyryl-carnitine are significantly increased both in humans with OCT1 deficiency [47,48] and in OCT1-knockout mice [49]. Although a direct transport of isobutyryl-carnitine by mouse OCT1 had initially been proposed [49], this could not be confirmed for human OCT1 [48]. These findings suggest that the increased circulating isobutyryl-carnitine levels are likely mediated by indirect, OCT1-dependent alterations in hepatic metabolic pathways. The precise mechanisms underlying these metabolic changes, however, remain to be elucidated.

Furthermore, we were unable to replicate most of the metabolic phenotypes previously reported for OCT1-knockout mice in our human cohorts. In mice, OCT1 deletion affects not only liver fat content but also multiple metabolic traits, including blood glucose levels, plasma triglycerides, and overall body fat content [18]. In contrast, in our analysis of analogous parameters in humans, only plasma triglycerides showed an association with OCT1 activity in both study cohorts. Even in this case, the effect size was small, and no consistent trend across genotypes (0 to 2 fully active alleles) could be observed (Supplementary Table S8). It cannot be excluded that the systemic metabolic phenotype observed in OCT1-knockout mice reflects not only the absence of OCT1 in the liver but also in the kidney.

It should be noted that the mouse data were generated through histological evaluation of liver tissue [17,18]. To date, liver biopsy remains the most precise method for quantifying hepatic fat content and assessing the histopathological features of fatty liver disease in humans. However, because it is invasive and associated with non-negligible risks, it is unsuitable for use in clinical studies without clear diagnostic indication [50]. PDFF measurement by MRI, a highly precise and non-invasive technique, is therefore the gold standard for assessing liver fat content in large population-based cohorts. PDFF correlates strongly with histopathologically assessed liver fat content (r = 0.89, [50]) and demonstrates high sensitivity and specificity for detecting hepatic steatosis (0.93 and 0.94, respectively [51]).

In our exploratory analysis of SHIP, we observed indications of a sex-dependent association between OCT1 deficiency and lower liver fat content (Table 2 and Table 4 and Supplementary Tables S6 and S7). Sex is a well-established determinant of hepatic steatosis: men generally show a higher prevalence of MASLD; whereas, the incidence increases sharply in post-menopausal women and exceeds that of men in the 60–69-year age group [52,53]. Sex hormones such as estrone, estradiol, estriol, and progesterone, have been reported to modulate OCT1 expression in vitro [54]. However, to date, no sex-specific effects of OCT1 deficiency in human and mice have been reported [19,55]. In the UK Biobank population, we observed sex-specific differences only in subgroups stratified by BMI and daily alcohol consumption. This pattern suggests that while certain metabolic conditions—such as increased body weight combined with low alcohol consumption—may modify the protective effect of OCT1 deficiency, no consistent subgroup-specific effects could be observed for sex, alcohol consumption, and being overweight.

In general, BMI remained the strongest predictor of liver fat content in our analyses (Table 3). This is consistent with established concepts of MASLD pathogenesis previous reports [56]. In addition to BMI, alcohol consumption, age, and sex were also significantly associated with liver fat content in our study population. Thus, prevention and treatment of obesity, along with lifestyle modification, continue to represent the most effective therapeutic strategies for patients with MASLD. However, these factors collectively explained only about 30% of the inter-individual variability in liver fat content in both cohorts analyzed. This highlights the importance of additional determinants, including genetic factors such as OCT1 and other loci reported previously, in identifying further mechanisms underlying hepatic fat accumulation and to develop targeted therapies for MASLD.

Our approach using population-based cohort studies has several limitations. First, our analyses focus solely on hepatic lipid accumulation measured by non-invasive MRI and do not include histopathological characterization of liver tissue. MASLD encompasses a histopathological spectrum ranging from simple steatosis to steatohepatitis and fibrosis, with higher fibrosis stages contributing disproportionately to disease burden [31,57]. Accurate staging, however, requires invasive procedures that are not feasible in epidemiological settings.

Then, although the association between genetically determined OCT1 deficiency and reduced liver fat content was replicated in two independent human cohorts and is consistent with findings in OCT1-knockout mice, the underlying molecular mechanisms remain unclear. To address this limitation, we performed complementary experiments in HEK293 cells. However, first mechanistic insights gained from this well-established cell model have to be regarded as preliminary, as its renal origin limits its ability to faithfully replicate hepatocellular metabolism and, most prominently, lipid accumulation. Further experimental work is needed to elucidate the metabolic pathways linking OCT1 function to hepatic fat storage.

Laboratory and imaging protocols differed between the two cohort studies, as well as within SHIP, necessitating cautious interpretation when comparing results across populations. To address this, we conducted all association analyses within each cohort study separately. Additionally, adjustment for the SHIP recruitment cohort did not substantially alter the observed associations (Supplementary Table S11). Nevertheless, residual bias between cohorts cannot be fully excluded.

Another limitation is that we did not adjust for genetic ancestry. As the frequency of genetically determined OCT1 deficiency varies across ethnic groups [28], ancestry-related bias cannot be excluded. However, several lines of evidence argue against substantial ancestry-related bias in our findings. First, the SHIP cohort has a highly homogeneous population structure and originates from a geographically restricted region in northeastern Germany. Second, more than 90% of UK Biobank participants are of European ancestry [58], and the OCT1 genotype distribution in the more genetically heterogeneous UK Biobank population was similar to that observed in SHIP (Supplementary Table S5). Third, and most importantly, individuals of Asian and African ancestry, who represent the next largest ancestry groups in the UK Biobank at approximately 2–3% each [58], have a lower or similar prevalence of MASLD [59]. However, because both populations also have a substantially lower frequency of OCT1-deficiency alleles [28], population stratification would be expected to bias the association toward an increased, rather than decreased, risk of MASLD among individuals with OCT1 deficiency.

As both studies are drawn from the general population, some participants may be receiving regular treatment with drugs such as metformin that affect glucose homeostasis and liver fat. We were unable to adjust for these medications in our analysis. However, our population-based cohorts confirm findings first reported in drug-naïve mice, in which OCT1 deficiency showed an even stronger association with reduced liver fat content [17]. We therefore consider it unlikely that drug exposure explains the observed association.

Finally, being overweight was assessed in this study using BMI measurements. BMI remains the most prominent surrogate marker for adiposity [31]. However, its use is controversially discussed, as other parameters such as fat mass index and waist-to-hip ratio show stronger associations with adiposity associated mortality [60,61]. The usage of BMI, however, provides larger comparability to other studies and guidelines addressing MASLD [31,45].

In conclusion, we demonstrate that genetically determined OCT1 deficiency is associated with reduced liver fat content in two large population-based cohort studies. The magnitude of this association is comparable to some previously established genetic determinants of fatty liver and is consistent with the effects on liver fat observed in OCT1-knockout mice. Together, these findings suggest further analysis of OCT1 as a potential risk factor for the pathogenesis of MASLD and a therapeutic target for early therapy or prevention of fatty liver.

## 4. Materials and Methods

### 4.1. Human Data

#### 4.1.1. Study Populations

The study was carried out using data from the Study of Health in Pomerania (SHIP, [29]) START and TREND cohorts and the UK Biobank. The SHIP was used as an exploratory cohort to create new hypotheses regarding the influence of subgroup stratification, while the UK Biobank was used to confirm these findings. The present analysis was conducted on all participants of both SHIP cohorts and in UK Biobank for whom a complete dataset including MRI quantified liver fat content, imputed OCT1 genotype and covariate predictor variables were obtained. A total of 2512 participants were included from SHIP and a further 31,594 from the UK Biobank.

Written informed consent was obtained from all volunteers of the SHIP study database before data assessment and the assessment was approved by the ethics committee of the University of Greifswald. The present study was approved by the data availability board of SHIP under the application number SHIP/2021/82/M+D. In the case of the UK Biobank, informed consent was given by its participants as well and the North West Multi-center Research Ethics Committee approved of the data collection. The present study was approved by the UK Biobanks Access Committee under the application number 319358. All data collection and the associated medical examinations were carried out in accordance with the Declaration of Helsinki and other relevant guidelines and regulations, ensuring both the quality of the data and the safety of the participants.

#### 4.1.2. SHIP

SHIP comprises a large database of adults, aged 20–79 years, with a wide portfolio of phenotypical features and genotyping data. Study design and sample selection of this ongoing population-based project is described elsewhere [29]. Two independent cohorts of SHIP were pooled as one cohort for the present study. In the years between 1997 and 2001, a two-stage stratified cluster sample was drawn from the total population of West Pomerania, forming a cohort of 4308 people. A total of 2332 of them enrolled in the second follow-up in the years 2008 to 2011. The participating subjects of this follow-up form the first cohort used for the present analysis (referred to as SHIP-START-2). In addition, a separate stratified random sample was drawn from the population in West Pomerania in the years between 2008 and 2012. This formed the second cohort used in the present analysis, with a total of 4420 participants (referred to as SHIP-TREND). Both cohorts of SHIP therefore stem from a limited region in northeastern Germany with a highly genetically homogeneous population structure. Written informed consent was obtained from all volunteers before study inclusion and the ethics committee of the University of Greifswald approved the SHIP-START-2 and SHIP-TREND (study protocol BB 39/08a). This population-based study was approved by the local institutional review board of SHIP (application number SHIP/2021/82/M+D).

#### 4.1.3. UK Biobank

We confirmed our findings from SHIP using the UK Biobank database. Study design and sample collection have been previously described elsewhere [30]. Data for the present study was obtained under the UK Biobank application number 319358. All data used in this research is publicly available to registered researchers through the UK Biobank data-access protocol. Data management of UK Biobank data was managed through a cloud-based platform, the research analysis platform (RAP). Phenotypic data was retrieved and filtered using the table exporter and a protocol available elsewhere [62].

#### 4.1.4. Whole-Body Magnetic Resonance Imaging

PDFF assessed by MRI was used to assess liver fat content in this study. It is highly reproducible across magnetic resonance (MR) systems and across imaging parameters [41,42,63].

Whole-body magnetic resonance imaging (WB-MRI) was performed in participants in both SHIP cohorts. The technique has already been described in detail [43]. In short, a standardized MRI protocol was followed using a 1.5-T MR imager (Magnetom Avanto; Siemens Medical Systems, Erlangen, Germany). WB-MRI was used for quantification of liver, pancreatic, and splenic fat content, as well as subcutaneous adipose tissue (SAT) and total visceral adipose tissue (VAT). Liver and pancreatic fat measurements were conducted according to Kühn et al. [64,65]. Liver fat content was assessed using chemical shift-encoded magnetic resonance imaging (CSE MRI). VAT and SAT were quantified as described elsewhere [65,66].

MRI examinations of the brain, heart and abdomen were performed on a subset of the UK Biobank study population. The MRI protocol has been previously described elsewhere [67,68,69].

#### 4.1.5. Covariates

Covariates included in this study comprised 19 baseline variables, including general epidemiological data (age, sex, daily alcohol consumption), data from physical examination (BMI, waist circumference, waist-to-height ratio), bioelectrical impedance analysis data (body fat percentage) and laboratory data from blood serum (albumin, alanine aminotransferase [ALAT], aspartate aminotransferase [ASAT], total bilirubin, FLI, gamma glutamyltransferase [GGT], fasting glucose, glycated hemoglobin [HbA1c], HDL cholesterol, LDL cholesterol, mean corpuscular hemoglobin [MCH], triglycerides). General epidemiological data was obtained by computer-assisted personal interviews for the SHIP population [29] and touchscreen questionnaires for the UK Biobank [30]. This study applied the term “sex” for biological sex as defined by chromosomal genotype and physiological features. It was categorized using the binary categories female and male and was self-reported by each participant. Daily alcohol consumption for the SHIP study population had previously been calculated based on self-reported drinking behavior in the past month. For the UK Biobank population, it was calculated for this study based on the self-reported drinking behavior in the past week or month, whichever was available. Average drink size and alcohol concentration [70] (Supplementary Table S1) were multiplied by the reported number of drinks of each beverage and divided by the number of days in each time period (7 days for a week and 30 days for a month). Calculated alcohol consumption was reported as grams of pure ethanol per day. Calculation and validation of the FLI has been described elsewhere [71,72]. Briefly, it is a prediction tool for hepatic steatosis using non-invasive biomarkers (serum triglycerides, serum GGT, BMI and waist circumference). Higher FLI values correlate with higher probability of hepatosteatosis [71,72]. In case of the UK Biobank all covariates were calculated using data from the second instance of assessment, as it was the date of the imaging visit, unless no data was available. In this case, data from instance 0 (first assessment) was used. This applied for the laboratory data.

#### 4.1.6. OCT1 Genotypes

Single nucleotide polymorphisms (SNPs) of interest were non-synonymous polymorphisms causing a functional amino acid substitution in OCT1. See Supplementary Table S2 for a detailed list of the analyzed SNPs and the resulting amino acid substitution included in this study. Based on these polymorphisms we calculated the six most common haplotypes that are characterized by one or a combination of these substitutions and used them further in the study (Supplementary Table S3). Haplotype and consequently genotype inference for each subject was done using the software Phase version 2.1.

For analysis of the association between genetically determined OCT1 deficiency and liver fat content, the genetically predicted OCT1 activity score was calculated. The substrate(s) underlying a potential association between OCT1 activity and liver fat content was unknown prior to this analysis. We therefore based the genetically predicted OCT1 activity score on OCT1 haplotypes, which were determined for each participant as described below. Each OCT1 allele was assigned an activity score ranging from 0 to 1 based on previously published in vitro data on the functional consequence of the respective OCT1 variant [23,28,73]. The activity scores of both OCT1 alleles were added for each participant, resulting in a genetically predicted OCT1 activity score ranging from 0 to 2. Our approach of deriving genetically predicted OCT1 activity scores from in vitro data has been widely applied in previous in vivo analyses investigating OCT1 pharmacogenetics [21,22,23,24,25,26,27,46].

The OCT1*1 allele exhibits full transport activity, while few to no remaining transport activity is described for alleles OCT1*3 through OCT1*6. We therefore assigned an activity score of 1 to the OCT1*1 reference allele and a score of 0 to the reduced-function alleles OCT1*3 through OCT1*6.

The second most common allele in Germany, OCT1*2, confers substrate-specific loss of OCT1 function [28]. Consequently, its activity score depends on the substrate considered and could theoretically range from 0 to 1. As the substrate underlying a possible connection of genetically predicted OCT1 deficiency and liver fat content remains unknown, no substrate-specific assignment was possible. Thiamine has been proposed previously as candidate substrate before [17], and OCT1*2 is described to have at least 50% of full in vitro transport activity for thiamine [46]. For the main analysis we therefore considered OCT1*2 to be a fully active allele (genetically predicted OCT1 activity score of 1). However, we performed exploratory analysis considering the *2 allele as fully active, half-active, and inactive (genetically predicted OCT1 activity score of 1, 0.5, and 0, respectively).

The SHIP-START-2 cohort was genotyped using the Affymetrix Human SNP Array 6.0 (Affymetrix, Santa Clara, CA, USA) following the manufacturer’s standard recommendations. Overall efficiency was 98.55%. The SHIP-TREND cohort was genotyped using the Illumina HumanOmni 2.5 for a subset of the cohort (n = 986). The final sample call rate was 99.51%. The remaining SHIP-TREND participants (n = 3133) were genotyped using Illumina Infinium Global Screening Array (San Diego, CA, USA).

Not all SNPs of interest were genotyped in both study cohorts. Therefore, genotype data was derived by standard imputation procedures using the software Eagle2 and Minimac3 against the 1000 Genomes Project Phase 3 Version 5 data for rs202220802 and the HRC (r1.1 2016) reference panel for remaining SNPs as implemented on the Michigan Imputation Server. See Supplementary Table S2 for observed-to-expected variance ratio (R2) as an indication for imputation quality. See Supplementary Table S4 for details on genotyping and imputation.

Previously available imputation data was used for the UK Biobank population. Genotyping and imputation of the UK Biobank have been described elsewhere [74].

### 4.2. In Vitro Data

Metabolic phenotype of OCT1-overexpressing cells was assessed using two Agilent Seahorse Assays. We compared HEK293 cells stably transfected with hOCT1 to cells transfected with the empty control vector (pcDNA5). The generation and characterization of these cell lines have been described previously [23,28,34]. Stable transfection resulted in strong and consistent expression of human OCT1 at the cell membrane. This was accompanied by significantly increased cellular uptake of characteristic OCT1 substrates, including thiamine, compared with control cells stably transfected with the empty vector [23,24,34].

In short, the assays were prepared according to the manufacturer’s protocol with the following modifications. Cells were then seeded at a final density of 4 × 10^4^ cells per well in wells previously coated with 50 µL poly-D-lysine one day prior to assay. On the day of the assay, XF Assay Medium was prepared by adding D-glucose, D-pyruvate and L-glutamine to XF Base Medium at a final concentration of 100 mg/mL, 10 mg/mL, and 200 mM, respectively. XF Base Medium contained thiamine*HCl based on Dulbecco’s Modified Eagle’s Medium (DMEM) at a concentration of 4 mg/L, as indicated by the manufacturer’s data sheet. The XFp Real-Time ATP Rate Assay Kit was used to assess the impact of hOCT on ATP production rates. Oligomycin was added to a final concentration of 1500 nM and Rot/AA to a final concentration of 500 nM. The XFp Agilent Seahorse Mito Stress Test Kit was used to assess the impact of hOCT1 on cellular respiration. Oligomycin, FCCP, and Rot/AA were added at final concentrations of 500 nM, 125 nM and 500 nM, respectively.

Both assays were additionally used to assess the impact of OCT1 overexpression on the metabolic phenotype under different energy sources. To this end, cells were starved for 1h prior to assay by adding XF Assay Medium without any energetic substrates. This has been described to enhance the cell’s responsiveness to specific cellular substrates [75]. During the assay, only XF Assay Medium without energetic substrates was used and one of the four energy sources of interest (D-glucose, L-glutamine, D-pyruvate, and oleic acid [OA]) was injected during the first injection cycle at final concentrations of 10 mM, 2 mM, 10 mM, and 200 µM, respectively, followed by five measurement cycles. Afterwards, the inhibitors of oxidative phosphorylation were injected as described previously.

D(+)-Glucose was obtained from Merck (Darmstadt, Germany); Sodium pyruvate was obtained from AppliChem (Darmstadt, Germany). High Glucose Dulbecco’s modified Eagle’s medium (DMEM), No Glucose No Glutamine DMEM, and fetal bovine serum (FBS) were obtained from Life Technologies (Darmstadt, Germany). Dulbecco’s phosphate-buffered saline (DPBS), and L-Glutamine (200 mM) were obtained from PAN-Biotech (Aidenbach, Germany). Poly-D-lysin (1–5 kDa) was obtained from Sigma-Aldrich. Seahorse materials and chemicals were obtained from Agilent (Santa Clara, CA, USA).

### 4.3. Statistical Analysis

To assess whether liver fat content is related to genetically predicted OCT1 activity, we tested the association of MRI-derived liver fat content in % of liver volume and genetically predicted OCT1 activity score. According to our main hypothesis, we tested whether liver fat content increases with increasing genetically predicted OCT1 activity score. Therefore, we used a one-sided non-parametric Jonckheere–Terpstra test, which accounted for a trend between each group of increasing genetically predicted OCT1 activity. We tested this hypothesis first in the SHIP population and then aimed to confirm the findings in the UK Biobank. Additionally, we performed multiple linear regression models to correct for age, sex, BMI and alcohol consumption. Liver fat content was natural-log transformed for regression analysis. Effect sizes were calculated as follows: effect size for the Jonckheere–Terpstra test was calculated as $r=\frac{z}{\sqrt{n}}$, where r is the effect size, z is the calculated test statistic and n is the number of participants. The explanatory strength of the multiple regression models was assessed using the explained variance of the model (R^2^), indicating the percentage of the inter-individual variance that could be predicted by the included parameters [76]. The effect of the genetically predicted OCT1 activity score on this prediction was assessed by calculation of the increase in this explanatory power ΔR^2^, which is calculated as ΔR2=R2(model with inclusion of OCT1)−R2(model without inclusion of OCT1).

In addition, we conducted exploratory analyses regarding confounding and mediating variables and the effect of genetically predicted OCT1 activity score on other metabolic parameters in the SHIP population. To this end, stratification was performed by sex, diagnosis of overweight (threshold: BMI ≥ 25 kg/m^2^) and low vs. high daily alcohol consumption (threshold: >10 g/d for women and >20 g/d for men) and increasing genetically predicted OCT1 activity score was tested for increasing liver fat content. Secondary outcomes included fat content in other body compartments (SAT, VAT, and pancreatic fat), clinically relevant blood parameters and fulfillment of the diagnostic criteria of MRI-defined hepatic steatosis. MRI-defined hepatic steatosis was defined as liver fat content >5.1% of liver volume based on the MRI data [45,65]. The Jonckheere–Terpstra test was applied to determine statistically significant differences in the primary and secondary outcomes between groups grouped by genetically predicted OCT1 activity score. Multiple logistic regression was applied to determine association of genetically predicted OCT1 activity score and diagnosis of MRI-defined hepatic steatosis. We then aimed to confirm all exploratory findings from the SHIP population in the population of the UK Biobank.

Raw data of OCR, ECAR and proton efflux rate (PER) from each measurement cycle of a Seahorse assay was obtained using the Wave desktop software, v1.1.1.3. Parameters of metabolic phenotype, ATP production rates, and mitochondrial respiration were calculated as described by the manufacturer. All measurements were normalized to the baseline OCR (mean OCR of measurements before oligomycin injection).

Statistical analysis was done using R (version 4.5.2, [77]), including packages haven (version 2.5.5), readxl (version 1.4.5), xtable (version 1.8-4), tidyverse (version 2.0.0, [78]), dplyr (version 1.1.4), ggplot2 (version 4.0.1, [79]), ggpubr (version 0.6.2), ggsignif (version 0.6.4), ggtext (version 0.1.2), circlize (version 0.4.17), greekLetters (version 1.0.4), showtext (version 0.9-7), RColorBrewer (version 1.1-3), rstatix (version 0.7.3, [80]) and DescTools (version 0.99.60) for data loading, manipulation, visualization, and statistical testing, respectively. All packages were obtained from the Comprehensive R Archive Network (CRAN) (https://cran.r-project.org/). Continuous data is expressed as median with interquartile range (IQR) as distribution parameter unless otherwise noted.

## Figures and Tables

**Table 1:** **Basic characteristics of the study population.** Shown are medians (IQR) unless otherwise noted.

Parameter	SHIP Study			UK Biobank		
	All	Women	Men	All	Women	Men
Number of participants (n)	2512	1279	1233	31,594	16,101	15,493
Age [y]	52 (42, 63)	52 (42, 62)	52 (42, 63)	66 (59, 71)	65 (59, 70)	66 (60, 72)
BMI [kg/m^2^]	27.2 (24.4, 30.3)	26.4 (23.3, 30.3)	27.7 (25.4, 30.4)	25.9 (23.5, 28.9)	25.3 (22.7, 28.6)	26.5 (24.4, 29.1)
Liver fat content [%]	4.01 (2.48, 8.30)	3.27 (2.22, 6.56)	5.05 (2.91, 9.45)	2.91 (2.03, 4.90)	2.62 (1.88, 4.20)	3.26 (2.24, 5.63)
MRI-defined hepatic steatosis ^1^	1017 (40.5%)	407 (31.8%)	610 (49.5%)	7502 (23.7%)	3070 (19.1%)	4432 (28.6%)
Fatty liver index	47.1 (20.7, 77.2)	29.5 (11.7, 62.9)	62.3 (36.6, 84.1)	39.5 (17.2, 68.8)	23.1 (10.1, 50.3)	56.3 (32.6, 77.9)
Alcohol consumption [g/d]	4.3 (1.3, 11.1)	2.2 (0.7, 5.4)	8.6 (2.8, 18.7)	11.0 (3.1, 22.5)	7.2 (1.6, 15.2)	16.5 (6.4, 30.0)
Body fat [%]	27.8 (22.9, 34.2)	33.9 (28.8, 38.4)	23.5 (20.1, 26.8)	30.6 (25.2, 37.0)	36.5 (31.9, 40.9)	25.9 (22.2, 29.5)
Waist-to-height ratio	0.52 (0.47, 0.58)	0.5 (0.45, 0.57)	0.54 (0.50, 0.59)	0.52 (0.48, 0.57)	0.5 (0.46, 0.56)	0.53 (0.49, 0.57)
Waist circumference [cm]	89.4 (80.0, 99.0)	82.5 (74.8, 92.2)	95.0 (88.0, 103.5)	89.0 (80.0, 98.0)	82.0 (75.0, 91.0)	94.0 (88.0, 101.0)

^1^ Threshold liver fat content >5.1% liver fat content. Numbers of individuals are shown, with percentages of the total subgroup in parentheses.

**Table 2:** **Association between genetically predicted OCT1 activity score and MRI-derived liver fat content.** Analyses were performed in the overall study population and stratified by sex. Medians (IQR) are shown.

Genetically Predicted OCT1 Activity Score	0		1		2			
Subgroup	n	Liver Fat Content ^1^	n	Liver Fat Content ^1^	n	Liver Fat Content ^1^	r	*p* Value
SHIP study								
Whole population	56	3.59 (4.65)	629	3.85 (4.78)	1827	4.07 (6.11)	0.038	0.027
Women	25	2.32 (1.91)	311	3.03 (3.04)	943	3.41 (5.2)	0.092	0.0005
Men	31	5.11 (7.34)	318	5.02 (6.28)	884	5.05 (6.64)	−0.006	0.589
UK Biobank								
Whole population	500	2.78 (2.26)	6871	2.83 (2.69)	24,223	2.95 (2.93)	0.026	2.86 × 10^−6^
Women	276	2.59 (2.11)	3602	2.57 (2.15)	12,223	2.64 (2.37)	0.021	0.0036
Men	224	3 (2.32)	3269	3.18 (3.36)	12,000	3.28 (3.42)	0.023	0.0024

The *p*-value was calculated using the Jonckheere–Terpstra test. ^1^ in % of liver volume.

**Table 3:** **Multiple linear regression analysis including genetically predicted OCT1 activity score as predictor for MRI-derived liver fat content.** Liver fat content was transformed by natural logarithm.

Model	Parameter	SHIP Study				UK Biobank			
		b	SE b	β	*p* Value	b	SE b	β	*p* Value
Common risk factors	Constant	−1.285	0.102		1.19 × 10^−35^	−1.034	0.039		6.49 × 10^−157^
	Age	0.009	0.001	0.158	2.02 × 10^−21^	0.004	0.000	0.042	2.19 × 10^−18^
	BMI	0.093	0.003	0.511	3.87 × 10^−177^	0.078	0.001	0.499	<1.0 × 10^−300^
	Daily alcohol consumption	0.005	0.001	0.078	6.12 × 10^−6^	0.003	0.000	0.091	1.83 × 10^−73^
	Sex	−0.173	0.028	−0.108	4.67 × 10^−10^	−0.091	0.007	−0.067	1.82 × 10^−39^
	*R* ^2^	0.36318				0.27923			
Common risk factors including OCT1	Constant	−1.471	0.122		1.18 × 10^−32^	−1.133	0.043		2.71 × 10^−150^
	Age	0.009	0.001	0.158	1.81 × 10^−21^	0.004	0.000	0.042	2.36 × 10^−18^
	BMI	0.093	0.003	0.510	4.87 × 10^−177^	0.078	0.001	0.499	<1.0 × 10^−300^
	Daily alcohol consumption	0.005	0.001	0.080	3.52 × 10^−6^	0.003	0.000	0.092	8.95 × 10^−74^
	Sex	−0.174	0.028	−0.108	3.73 × 10^−10^	−0.091	0.007	−0.066	6.76 × 10^−39^
	Genetically predicted OCT1 activity score	0.07	0.026	0.044	6.01 × 10^−3^	0.036	0.007	0.024	3.77 × 10^−7^
	Δ*R*^2 +^	0.00192				0.00059			

SE, standard error. ^+^ Change in explanatory power of the model after inclusion of OCT1 as risk factor.

**Table 4:** **Association of genetically predicted OCT1 activity score and MRI-derived liver fat content.** Analyses were performed on the overall study population and stratified by sex. Examination of liver fat content was further stratified for common risk factors of hepatic steatosis. Shown are median (IQR).

Genetically Predicted OCT1 Activity Score	0		1		2			
Subgroup	n	Liver Fat Content ^1^	n	Liver Fat Content ^1^	n	Liver Fat Content ^1^	r	*p* Value
**SHIP study**								
	**Whole population**							
Low daily alcohol	44	3.12 (4.69)	519	3.63 (4.38)	1529	3.97 (5.85)	0.051	0.0101
High daily alcohol	12	3.77 (2.51)	110	5.26 (5.92)	298	5.33 (7.31)	−0.005	0.5414
Non-overweight	20	2.27 (2.18)	185	2.36 (1.26)	551	2.53 (1.54)	0.083	0.0109
Overweight	36	4.71 (6.93)	444	5.11 (6.22)	1276	5.69 (8.17)	0.040	0.0457
	**Women**							
Low daily alcohol	22	2.19 (2.08)	280	3.05 (3.05)	834	3.49 (5.32)	0.100	0.0004
High daily alcohol	3	3.70 (0.24)	31	2.93 (2.91)	109	2.92 (3.92)	0.027	0.3748
Non-overweight	12	2.11 (0.81)	119	2.28 (1.09)	365	2.46 (1.52)	0.121	0.0034
Overweight	13	3.36 (2.96)	192	4.14 (4.45)	578	5.01 (8.10)	0.110	0.0011
	**Men**							
Low daily alcohol	22	6.01 (9.16)	239	4.62 (6.01)	695	4.53 (6.15)	−0.004	0.5491
High daily alcohol	9	5.09 (4.82)	79	6.71 (7.18)	189	6.52 (7.95)	0.016	0.3962
Non-overweight	8	4.05 (2.05)	66	2.66 (1.59)	186	2.73 (1.89)	0.024	0.3520
Overweight	23	7.68 (10.17)	252	6.29 (6.43)	698	6.29 (8.32)	−0.014	0.6743
**UK Biobank**								
	**Whole population**							
Low daily alcohol	288	2.69 (2.31)	3946	2.76 (2.58)	13,922	2.85 (2.81)	0.020	0.0042
High daily alcohol	212	2.84 (2.15)	2925	2.93 (2.82)	10,301	3.09 (3.08)	0.034	4.42 × 10^−5^
Non-overweight	196	2.23 (1.25)	2754	2.22 (1.24)	9689	2.25 (1.35)	0.016	0.0381
Overweight	304	3.34 (3.39)	4117	3.62 (4.07)	14,534	3.79 (4.31)	0.032	4.68 × 10^−6^
	**Women**							
Low daily alcohol	165	2.50 (2.3)	2054	2.57 (2.21)	6995	2.62 (2.50)	0.022	0.0162
High daily alcohol	111	2.72 (1.97)	1548	2.59 (2.03)	5228	2.66 (2.24)	0.020	0.0522
Non-overweight	125	2.03 (1.21)	1703	2.14 (1.19)	5774	2.12 (1.21)	−0.001	0.5406
Overweight	151	3.22 (3.68)	1899	3.25 (3.4)	6449	3.52 (4.02)	0.042	5.48 × 10^−5^
	**Men**							
Low daily alcohol	123	3.10 (2.45)	1892	2.99 (3.18)	6927	3.08 (3.09)	0.011	0.1446
High daily alcohol	101	2.92 (2.23)	1377	3.53 (3.56)	5073	3.62 (3.81)	0.038	0.0010
Non-overweight	71	2.36 (1.25)	1051	2.34 (1.36)	3915	2.47 (1.46)	0.035	0.0068
Overweight	153	3.39 (2.91)	2218	4.00 (4.64)	8085	4.01 (4.47)	0.021	0.0172

The *p*-value was calculated using the Jonckheere–Terpstra test. ^1^ in % of liver volume.

## Data Availability

The datasets analyzed in the current study were obtained from the Community Medicine Research network of the University of Greifswald, Germany and the UK Biobank database. Availability of the data restricted to registered studies only, and the data was used under license for the current study. The dataset of the Study of Health in Pomerania is available after application to the Community Medicine Research network of the University of Greifswald, Germany. The UK Biobank dataset is available following application to the UK Biobank Access Management System. The authors had no special access privileges, and other researchers may obtain all datasets using the same application procedures.

## Supplementary Materials

The following supporting information can be downloaded at: https://www.mdpi.com/article/10.3390/ijms27167422/s1.

## Abbreviations

The following abbreviations are used in this manuscript:

ALAT	Alanine aminotransferase
ASAT	Aspartate aminotransferase
ATLAS	Automatic tissue and labeling analysis software
BMI	Body mass index
CCR2/CCR5	Chemokine receptor type 2/type 5
CI	Confidence interval
ECAR	Extracellular acidification rate
FGF21	Fibroblast growth factor 21
FLI	Fatty liver index
FXR	Farnesoid X receptor
GGT	Gamma-glutamyl transferase
HbA1c	Glycated hemoglobin
hOCT1	Human organic cation transporter 1
IQR	Interquartile range
MASLD	Metabolic dysfunction-associated steatotic liver disease
MCH	Mean corpuscular hemoglobin
MR	Magnetic resonance
MRI	Magnetic resonance imaging
OCR	Oxygen consumption rate
OCT1	Organic cation transporter 1
OR	Odds ratio
PER	Proton efflux rate
PDFF	Proton density fat fraction
PNPLA3	Patatin-like phospholipase domain-containing protein 3
RAP	Research analysis platform
SAT	Subcutaneous adipose tissue
SE	Standard error
SHIP	Study of Health in Pomerania
SNP	Single nucleotide polymorphisms
THRβ	Thyroid hormone receptor beta
THTR-1	Thiamine transporter 1
THTR-2	Thiamine transporter 2
TM6SF2	Transmembrane 6 superfamily member 2
VAT	Visceral adipose tissue
WB-MRI	Whole-body magnetic resonance imaging
