# Supplementary material for: Genetically Determined Loss-of-Function of the Organic Cation Transporter OCT1 Is Associated with Lower Liver Fat Content in Humans
Source: Int J Mol Sci. 2026 Aug 19;27(16):7422. doi: 10.3390/ijms27167422 (PMC13513528; doi:10.3390/ijms27167422)
Supplement: Supplementary file 1 [file ijms-27-07422-s001.zip › ijms-4423167-supplementary.pdf]

Supplementary information

Supplementary Table S1 - Categories of alcoholic beverages in UK Biobank*. Average volume and alcohol concentration was assessed according to British NHS [67]*

Beverage	Standard volume [mL]	Alcohol by volume [%]	Alcohol mass per drink [g]
Red wine	125	13.5	13.3
White wine	125	12.0	11.8
Beer	568	5.0	22.42
Fortified wine	80	20.0	12.63
Spirits	40	40.0	12.63
Others	275	4.6	9.98

***Supplementary Table S2** - **OCT1 single nucleotide polymorphisms (SNP) of interest** Each SNP resulted in an amino acid substitution or deletion that resulted in a loss-of-OCT1 function. Imputation quality is described as R^2 for both study cohorts. Directly genotyped SNPs are marked as such.*

**SNP**	**Amino acid substitution**	**R^2 in SHIP-START-2**	**R^2 in SHIP-TREND**
rs202220802	Met420del	0.99229	0.99098
rs12208357	Arg61Cys	0.97582	genotyped
rs34130495	Gly401Ser	0.91072	genotyped
rs55918055	Cys88Arg	0.79051	0.89434
rs34059508	Gly465Arg	0.88217	Genotyped

Supplementary Table S3 - Amino acid substitution or deletion and resulting OCT1 haplotypes
Single amino acid substitution or deletion and combinations resulted in the six most common OCT1 haplotypes in Europe included in this study.

**OCT1 haplotype**	**Arg61Cys**	**Cys88Arg**	**Gly401Ser**	**Met420del**	**Gly465Arg**
****1***	Arg	Cys	Gly	Met	Gly
****2***	Arg	Cys	Gly	**Del**	Gly
****3***	**Cys**	Cys	Gly	Met	Gly
****4***	Arg	Cys	**Ser**	Met	Gly
****5***	Arg	Cys	Gly	**Del**	**Arg**
****6***	Arg	**Arg**	Gly	**Del**	Gly

Supplementary Table S4 - Genotyping and imputation in both cohorts of SHIP

**Study cohort**	**Genotyping chip**	**Number of participants**	**Calling algorithm**	**SNP exclusions**	**Sample exclusion**	**Number of samples**	**SNP count**	**Prephasing / Imputation**
				(pre imputation QC)		(post imputation QC)		
SHIP-START	Affymetrix Genome-Wide Human SNP Array 6.0	4,308	Birdseed2	pHWE<10^{-4}<br>or<br>call rate<95%<br>or<br>monomorphic SNVs	call rate<92%<br>or<br>PCA outliers<br>or<br>duplicate samples (by IBS)<br>or<br>gender mismatch (reported / genotyped)	4,069	823,635	Imputation performed using Eagle v2.3 and Minimac3 against the 1000G Phase 3 v5 reference panel[1] or the HRC (r1.1 2016) reference panel[2] on the Michigan Imputation server
SHIP-TREND	Illumina Infinium HumanOmni2.5 BeadChip	4,420	GenomeStudio Genotyping Module v1.0 (GenCall)	pHWE<10^{-4}<br>or<br>call rate<95%<br>or<br>monomorphic SNVs	call rate<94%<br>or<br>PCA outliers<br>or<br>duplicate samples (by IBS)<br>or<br>gender mismatch (reported / genotyped)	986	1,803,558	
	Illumina Infinium Global Screening Array		GenomeStudio 2.0 Genotyping Module (GenCall)	pHWE<10^{-4}<br>or<br>call rate<95%<br>or<br>MAC<10<br>or<br>MAF<0.01	call rate<94%<br>or<br>PCA outliers<br>or<br>duplicate samples (by IBD)<br>or<br>gender mismatch (reported / genotyped) and heterozygosity ±4 sd from the mean	3,133	489,014	

[1] for rs202220802

[2] for rs12208357, rs34130495, rs55918055 and rs34059508

HRC, Haplotype Reference Consortium; HWE, Hardy-Weinberg-Equilibrium; IBD, identity by descent; IBS, identity by state; MAC, minor allele count; MAF, minor allele frequency; PCA, principal component analysis; QC, quality control; SHIP, Study of Health in Pomerania; SNP, single nucleotide polymorphism; SNV, single nucleotide variant

Supplementary Table S5 - OCT1 genotype distribution in study populations

**Study population**	**SHIP**	**UK Biobank**
**Number of participants**	**2,512**	**31,594**
**OCT1 genotype**	**n (%)**	**n (%)**
**1*	3,501 (69.7)	45,502 (72.0)
**2*	782 (15.6)	9,815 (15.5)
**3*	489 (9.7)	4,244 (6.7)
**4*	118 (2.3)	1,782 (2.8)
**5*	123 (2.5)	1,644 (2.6)
**6*	11 (0.2)	201 (0.3)

Supplementary Table S6 - Multiple linear regression analysis including genetically predicted OCT1 activity score as predictor for MRI-derived liver fat content in women and men.

Liver fat content was transformed by natural logarithm.

Model	Parameter	SHIP study				UK Biobank			
		**b**	**SE b**	**β**	**P value**	**b**	**SE b**	**β**	**P value**
**Women**									
Common risk factors	Constant	-1.430	0.113		$1.57x10^{-34}$	-1.385	0.048		$3.75x10\text{-}^{180}$
	Age	0.014	0.001	0.223	$1.53x10^{-20}$	0.008	0.001	0.089	$8.13x10^{-40}$
	BMI	0.078	0.004	0.488	$1.03x10^{-81}$	0.074	0.001	0.520	$<1.0x10^{-300}$
	Daily alcohol consumption	0.000	0.003	-0.002	0.928	0.004	0.000	0.073	$1.48x10^{-27}$
	R2	*0.34944*				*0.27768*			
Common risk factors including OCT1	Constant	-1.729	0.146		$1.12x10^{-30}$	-1.488	0.054		$1.24x10^{-160}$
	Age	0.014	0.001	0.222	$1.98x10^{-20}$	0.008	0.001	0.089	$7.36x10^{-40}$
	BMI	0.078	0.004	0.484	$3.92x10^{-81}$	0.074	0.001	0.521	$<1.0x10^{-300}$
	Daily alcohol consumption	0.000	0.003	-0.002	0.917	0.004	0.000	0.073	$1.30x10^{-27}$
	Genetically predicted OCT1 activity score	0.117	0.036	0.072	$1.33x10^{-3}$	0.038	0.010	0.026	$7.89x10^{-5}$
	ΔR2⁺	*0.00524*				*0.00070*			
**Men**									
Common risk factors	Constant	-1.935	0.142		$1.24x10^{-39}$	-1.042	0.054		$3.57x10\text{-}^{83}$
	Age	0.005	0.001	0.098	$3.18x10^{-5}$	0.000	0.001	-0.003	0.705
	BMI	0.117	0.005	0.553	$3.97x10^{-101}$	0.085	0.001	0.486	$<1.0x10^{-300}$
	Daily alcohol consumption	0.005	0.001	0.109	$2.35x10^{-6}$	0.003	0.000	0.097	$3.77x10^{-44}$
	R2	*0.3562*				*0.25497*			
Common risk factors including OCT1	Constant	-2.027	0.173		$3.86x10^{-30}$	-1.134	0.061		$1.89x10^{-77}$
	Age	0.005	0.001	0.099	$3.02x10^{-5}$	0.000	0.001	-0.003	0.692
	BMI	0.117	0.005	0.554	$3.34x10^{-101}$	0.085	0.001	0.486	$<1.0x10^{-300}$
	Daily alcohol consumption	0.005	0.001	0.110	$1.87x10^{-6}$	0.003	0.000	0.098	$2.28x10^{-44}$
	Genetically predicted OCT1 activity score	0.033	0.035	0.021	0.354	0.033	0.010	0.023	$1.10x10^{-3}$
	ΔR2⁺	*0.00045*				*0.00051*			

SE, standard error; + Change in explanatory power of the model after inclusion of OCT1 as risk factor

Supplementary Table S7 - Multiple logistic regression analysis including genetically predicted OCT1 activity score as predictor of MRI-defined hepatic steatosis. *Threshold for diagnosis of MRI-defined hepatic steatosis was a liver fat content>5.1%.*

Model	Parameter	SHIP study					UK Biobank				
		β	SE β	OR	95% CI OR	P value	β	SE β	OR	95% CI OR	P value
**Whole population**											
Common risk factors	Constant	-9.293	0.47			5.52x10^{-87}	-8.38	0.186			<1.0x10^{-300}
	Age	0.029	0.004	1.03	(1.02; 1.04)	2.00x10^{-15}	0.006	0.002	1.01	(1.00; 1.01)	1.68x10^{-3}
	BMI	0.289	0.014	1.34	(1.3; 1.37)	1.21x10^{-91}	0.262	0.004	1.30	(1.29; 1.31)	<1.0x10^{-300}
	Daily alcohol consumption	0.017	0.004	1.02	(1.01; 1.03)	3.16x10^{-05}	0.009	0.001	1.01	(1.01; 1.01)	4.26x10^{-33}
	Sex	-0.61	0.105	0.54	(0.44; 0.67)	7.05x10^{-9}	-0.388	0.032	0.68	(0.64; 0.72)	5.95x10^{-34}
Common risk factors including OCT1	Constant	-9.838	0.546			1.69x10^{-72}	-8.737	0.208			<1.0x10^{-300}
	Age	0.03	0.004	1.03	(1.02; 1.04)	1.68x10^{-15}	0.006	0.002	1.01	(1.00; 1.01)	1.69x10^{-3}
	BMI	0.289	0.014	1.34	(1.3; 1.37)	1.53x10^{-91}	0.262	0.004	1.30	(1.29; 1.31)	<1.0x10^{-300}
	Daily alcohol consumption	0.018	0.004	1.02	(1.01; 1.03)	2.22x10^{-5}	0.009	0.001	1.01	(1.01; 1.01)	2.78x10^{-33}
	Sex	-0.614	0.105	0.54	(0.44; 0.67)	5.70x10^{-9}	-0.385	0.032	0.68	(0.64; 0.72)	1.76x10^{-33}
	Genetically predicted OCT1 activity score	0.199	0.098	1.22	(1.01; 1.48)	0.043	0.126	0.033	1.13	(1.06; 1.21)	1.08x10^{-4}
**Women**											
Common risk factors	Constant	-10.381	0.619			3.36x10^{-63}	-10.032	0.266			<1.0x10^{-300}
	Age	0.047	0.006	1.05	(1.04; 1.06)	2.00x10^{-14}	0.025	0.003	1.03	(1.02; 1.03)	9.56x10^{-16}
	BMI	0.251	0.018	1.29	(1.24; 1.33)	5.62x10^{-46}	0.249	0.005	1.28	(1.27; 1.3)	<1.0x10^{-300}
	Daily alcohol consumption	0.019	0.012	1.02	(1; 1.04)	0.117	0.013	0.002	1.01	(1.01; 1.02)	4.60x10^{-13}
Common risk factors including OCT1	Constant	-11.28	0.764			2.56x10^{-49}	-10.471	0.302			2.61x10^{-263}
	Age	0.047	0.006	1.05	(1.04; 1.06)	1.67x10^{-14}	0.025	0.003	1.03	(1.02; 1.03)	7.43x10^{-16}
	BMI	0.25	0.018	1.28	(1.24; 1.33)	9.85x10^{-46}	0.25	0.005	1.28	(1.27; 1.3)	<1.0x10^{-300}
	Daily alcohol consumption	0.02	0.012	1.02	(1; 1.04)	0.106	0.013	0.002	1.01	(1.01; 1.02)	3.58x10^{-13}
	Genetically predicted OCT1 activity score	0.329	0.155	1.39	(1.02; 1.88)	0.034	0.154	0.049	1.17	(1.06; 1.28)	1.64x10^{-3}
**Men**											
Common risk factors	Constant	-10.988	0.706			1.21x10^{-54}	-8.445	0.246			8.12x10^{-259}
	Age	0.019	0.005	1.02	(1.01; 1.03)	1.00x10^{-4}	-0.007	0.003	0.99	(0.99; 1)	0.010
	BMI	0.349	0.024	1.42	(1.35; 1.49)	9.32x10^{-49}	0.282	0.006	1.33	(1.31; 1.34)	<1.0x10^{-300}
	Daily alcohol consumption	0.018	0.005	1.02	(1.01; 1.03)	9.68x10^{-5}	0.009	0.001	1.01	(1.01; 1.01)	6.11x10^{-22}
	Constant	-11.374	0.808			5.40x10^{-45}	-8.74	0.274			1.58x10^{-222}
	Age	0.019	0.005	1.02	(1.01; 1.03)	9.68x10^{-5}	-0.007	0.003	0.99	(0.99; 1)	0.009

Common risk factors including OCT1	BMI	0.35	0.024	1.42	(1.35; 1.49)	8.57×10^{-49}	0.282	0.006	1.33	(1.31; 1.34)	$<1.0 \times 10^{-300}$
	Daily alcohol consumption	0.018	0.005	1.02	(1.01; 1.03)	7.99×10^{-5}	0.009	0.001	1.01	(1.01; 1.01)	4.97×10^{-22}
	Genetically predicted OCT1 activity score	0.131	0.13	1.14	(0.88; 1.47)	0.314	0.108	0.044	1.11	(1.02; 1.21)	0.014

SE, standard error; OR, odds ratio; CI, confidence interval

Supplementary Table S8 - Association of genetically predicted OCT1 activity score and parameters of metabolic dysfunction. *Shown are median (IQR).*

**Genetically predicted OCT1 activity score**	**0**	**1**	**2**	**P value**
**SHIP study**				
ALAT[1]	0.42 (0.26)	0.38 (0.24)	0.38 (0.24)	0.760
Albumin [g/L]	41.0 (4.25)	40.0 (4.00)	40.0 (4.00)	0.425
ASAT[1]	0.30 (0.16)	0.30 (0.14)	0.29 (0.13)	0.513
Body fat [%]	27.9 (8.53)	27.6 (11.10)	27.8 (11.50)	0.314
Bilirubin (total) [µmol/L]	8.10 (4.53)	7.30 (3.70)	7.60 (4.10)	0.094
Cholesterol (total) [mmol/L]	5.60 (1.25)	5.50 (1.50)	5.40 (1.40)	0.432
Fatty liver index	42.1 (54.2)	47.8 (55.9)	46.9 (56.9)	0.774
GGT[1]	0.56 (0.34)	0.50 (0.30)	0.50 (0.32)	0.566
Glucose (fasting) [mmol/L]	5.50 (0.90)	5.40 (0.90)	5.40 (0.90)	0.546
HbA1c[2]	5.30 (0.60)	5.30 (0.70)	5.20 (0.70)	0.446
HDL cholesterol [mmol/L]	1.51 (0.56)	1.40 (0.52)	1.42 (0.52)	0.358
LDL cholesterol [mmol/L]	3.34 (1.45)	3.39 (1.35)	3.34 (1.16)	0.631
Waist circumference [cm]	87.5 (15.1)	89.5 (20.0)	89.5 (19.0)	0.975
Triglycerides [mmol/L][3]	1.32 (1.44)	1.41 (1.07)	1.35 (1.04)	0.032
Waist-to-height-ratio	0.52 (0.09)	0.53 (0.11)	0.52 (0.11)	0.993
**UK Biobank**				
ALAT[1]	19.4 (11.5)	19.7 (11.9)	19.9 (11.8)	0.386
Albumin [g/L]	45.4 (3.14)	45.3 (3.29)	45.4 (3.37)	0.013
ASAT[1]	23.9 (7.00)	24.1 (7.65)	24.3 (7.80)	0.027
Body fat [%]	31.2 (12.7)	30.9 (11.8)	30.5 (11.8)	0.005
Bilirubin (total) [µmol/L]	8.19 (4.02)	8.25 (3.96)	8.36 (4.07)	0.002
Cholesterol (total) [mmol/L]	5.69 (1.37)	5.71 (1.41)	5.66 (1.42)	0.000
Fatty liver index	40.8 (51.2)	39.2 (51.7)	39.5 (51.5)	0.885
GGT[1]	24.6 (19.6)	24.5 (19.4)	24.9 (20.2)	0.013
Glucose (fasting) [mmol/L]	4.90 (0.63)	4.86 (0.67)	4.88 (0.66)	0.236
HbA1c[2]	34.0 (4.82)	34.6 (4.80)	34.6 (4.90)	0.045
HDL cholesterol [mmol/L]	1.37 (0.47)	1.43 (0.49)	1.42 (0.51)	0.741
LDL cholesterol [mmol/L]	3.54 (1.10)	3.56 (1.10)	3.53 (1.11)	<0.001
Waist circumference [cm]	88.5 (15.0)	89.0 (17.0)	89.0 (18.0)	0.126
Triglycerides [mmol/L][3]	135.2 (107.0)	124.8 (90.4)	122.9 (91.2)	0.007
Waist-to-height-ratio	0.52 (0.08)	0.52 (0.09)	0.52 (0.09)	0.754

P value is calculated using the Jonckheere-Terpstra test.

[1] [μkatal/L] for SHIP study and [U/L] for UK Biobank

[2] [%] for SHIP study and [mmol/mol] for UK Biobank

[3] [mmol/L] for SHIP study and [mg/dL] for UK Biobank

Supplementary Table S9 - Association of genetically predicted OCT1 activity score and fat compartments measured by MRI. *Analyses were performed on the overall study population and stratified by sex. Shown are median (IQR).*

**Genetically predicted OCT1 activity score**	**0**		**1**		**2**			
**Fat compartment**	**n**	**Fat content1**	**n**	**Fat content**	**n**	**Fat content**	**r**	**P value**
**SHIP study**								
Whole population								
Subcutaneous fat [L]	55	6.83 (3.53)	587	7.19 (4.39)	1,708	7.19 (4.38)	0.008	0.6895
Visceral fat [L]	55	3.61 (3.95)	588	3.76 (3.52)	1,708	3.66 (3.79)	-0.004	0.8529
Pancreatic fat [%]	48	5.94 (3.34)	480	6.96 (4.03)	1,377	6.77 (3.99)	-0.004	0.8460
Women								
Subcutaneous fat [L]	25	7.49 (3.81)	281	8.30 (4.77)	869	8.22 (5.05)	0.024	0.4164
Visceral fat [L]	25	1.57 (1.44)	282	2.47 (2.67)	869	2.40 (2.71)	0.053	0.0697
Pancreatic fat [%]	22	5.53 (1.31)	242	6.38 (3.93)	724	6.19 (3.59)	0.002	0.9377
Men								
Subcutaneous fat [L]	30	6.57 (3.15)	306	6.47 (3.56)	839	6.32 (3.49)	-0.025	0.3875
Visceral fat [L]	30	5.51 (2.99)	306	5.24 (3.85)	839	5.22 (3.74)	-0.025	0.3842
Pancreatic fat [%]	26	7.26 (3.79)	238	7.52 (3.95)	653	7.51 (4.23)	0.001	0.9689
**UK Biobank**								
Whole population								
Subcutaneous fat [L]	500	6.38 (4.14)	6,866	6.25 (3.76)	24,196	6.23 (3.77)	-0.007	0.2299
Visceral fat [L]	500	3.44 (2.79)	6,868	3.33 (3.17)	24,207	3.38 (3.19)	0.007	0.2241
Pancreatic fat [%]	313	7.96 (9.17)	4,208	8.14 (8.93)	14,937	8.03 (8.84)	-0.003	0.6617
Women								
Subcutaneous fat [L]	276	7.76 (4.51)	3,602	7.35 (4.24)	12,211	7.38 (4.25)	-0.001	0.9375
Visceral fat [L]	276	2.62 (2.21)	3,601	2.38 (2.04)	12,214	2.38 (2.06)	-0.007	0.3744
Pancreatic fat [%]	174	6.21 (6.28)	2,208	6.38 (6.69)	7,524	6.15 (6.53)	-0.014	0.1769
Men								
Subcutaneous fat [L]	224	5.17 (2.54)	3,264	5.38 (2.73)	11,985	5.35 (2.71)	0.002	0.7590
Visceral fat [L]	224	4.62 (2.64)	3,267	4.79 (3.26)	11,993	4.79 (3.26)	0.001	0.8639
Pancreatic fat [%]	139	10.67 (9.42)	2,000	10.30 (10.26)	7,413	10.33 (10.22)	-0.006	0.5618

P-value is calculated using the Jonckheere-Terpstra test

Supplementary Table S10 - Parameters of ATP production rate and mitochondrial respiration in HEK293 cells

Parameter	hOCT1		Control		n	t statistic	p value[+]
	Mean	SEM	Mean	SEM			
**Standard medium**							
OCR/ECAR ratio	1.99	0.13	2.06	0.07	18	-0.488	1.000
Non-Mitochondrial Oxygen Consumption	0.26	0.01	0.28	0.01	18	-0.762	1.000
Basal Respiration	0.74	0.01	0.72	0.01	18	0.762	1.000
ATP-Production Coupled Respiration	0.41	0.04	0.56	0.01	9	-2.789	0.210
Proton Leak	0.32	0.04	0.19	0.01	9	2.256	0.529
mitoATP Production Rate	1.05	0.13	1.69	0.06	9	-3.077	0.103
glycoATP Production Rate	3.21	0.23	2.98	0.09	9	0.651	1.000
totalATP Production Rate	4.26	0.28	4.67	0.07	9	-1.030	1.000
Maximal Respiration	1.09	0.02	1.09	0.02	9	-0.053	1.000
Spare Respiratory Capacity	47.74	2.91	58.45	3.98	9	-1.536	1.000
**D-glucose**							
**OCR/ECAR ratio**	**0.94**	**0.05**	**0.76**	**0.02**	**20**	**3.256**	**0.033**
Non-Mitochondrial Oxygen Consumption	0.18	0.01	0.18	0.00	20	-0.032	1.000
**Basal Respiration**	**0.35**	**0.01**	**0.30**	**0.01**	**20**	**3.282**	**0.024**
ATP-Production Coupled Respiration	0.21	0.02	0.18	0.01	10	1.102	1.000
Proton Leak	0.15	0.01	0.11	0.01	10	2.153	0.509
mitoATP Production Rate	0.29	0.15	0.22	0.09	10	0.287	1.000
glycoATP Production Rate	3.41	0.14	4.14	0.13	10	-2.697	0.148
totalATP Production Rate	3.70	0.12	4.36	0.14	10	-2.521	0.215
Maximal Respiration	0.31	0.02	0.22	0.02	10	2.335	0.318
Spare Respiratory Capacity	-9.81	5.13	-29.20	6.72	10	1.622	1.000
**L-glutamine**							
OCR/ECAR ratio	14.58	0.63	13.78	0.70	10	0.841	1.000
Non-Mitochondrial Oxygen Consumption	0.21	0.00	0.22	0.01	10	-0.683	1.000
Basal Respiration	0.67	0.01	0.68	0.01	10	-0.185	1.000
ATP-Production Coupled Respiration	0.20	0.03	0.38	0.02	5	-3.587	0.123
Proton Leak	0.47	0.04	0.28	0.01	5	3.231	0.279
mitoATP Production Rate	0.36	0.08	1.02	0.11	5	-3.399	0.103
glycoATP Production Rate	0.15	0.02	0.19	0.03	5	-1.032	1.000
totalATP Production Rate	0.50	0.09	1.22	0.13	5	-3.171	0.152
Maximal Respiration	0.77	0.01	0.73	0.02	5	1.355	1.000
Spare Respiratory Capacity	12.45	1.35	5.36	1.33	5	2.642	0.296
**Oleic acid**							
OCR/ECAR ratio	11.69	0.23	12.37	0.24	8	-2.040	0.608
Non-Mitochondrial Oxygen Consumption	0.19	0.01	0.19	0.01	8	0.212	1.000
Basal Respiration	0.63	0.01	0.63	0.01	8	-0.318	1.000

ATP-Production Coupled Respiration	0.44	0.01	0.46	0.01	4	-1.045	1.000
Proton Leak	0.17	0.01	0.16	0.01	4	0.790	1.000
mitoATP Production Rate	1.44	0.02	1.48	0.05	4	-0.563	1.000
glycoATP Production Rate	0.14	0.01	0.12	0.01	4	0.970	1.000
totalATP Production Rate	1.57	0.02	1.59	0.04	4	-0.291	1.000
Maximal Respiration	0.47	0.04	0.41	0.03	4	0.876	1.000
Spare Respiratory Capacity	-27.19	4.78	-37.86	3.87	4	1.228	1.000
**Pyruvate**							
OCR/ECAR ratio	10.04	0.31	9.68	0.27	12	0.874	1.000
Non-Mitochondrial Oxygen Consumption	0.22	0.01	0.22	0.01	12	-0.300	1.000
Basal Respiration	0.75	0.01	0.77	0.01	12	-0.619	1.000
ATP-Production Coupled Respiration	0.33	0.08	0.42	0.06	6	-0.628	1.000
Proton Leak	0.42	0.08	0.33	0.06	6	0.690	1.000
mitoATP Production Rate	0.94	0.36	1.29	0.27	6	-0.538	1.000
glycoATP Production Rate	0.27	0.03	0.27	0.02	6	-0.065	1.000
totalATP Production Rate	1.22	0.34	1.56	0.26	6	-0.573	1.000
Maximal Respiration	0.63	0.07	0.62	0.06	6	0.076	1.000
Spare Respiratory Capacity	-16.84	9.41	-19.74	9.03	6	0.157	1.000

SEM – Standard error of mean; OCR – oxygen consumption rate; ECAR – extracellular acidification rate; ATP – adenosine triphosphate

[+] Unpaired Welch's t-Test for differences between hOCT1 overexpressing cells and control, Bonferroni correction for multiple testing within group of energy source

Supplementary Table S11 - Multiple linear regression analysis including genetically predicted OCT1 activity score as predictor for MRI-derived liver fat content in SHIP, adjusted for SHIP recruitment cohort.

Liver fat content was transformed by natural logarithm.

**Model**	**Parameter**	**SHIP study**			
		**b**	**SE b**	**β**	**P value**
Common risk factors	Constant	-1.208	0.116		5.64×10^{-25}
	Age	0.009	0.001	0.155	2.18×10^{-20}
	BMI	0.093	0.003	0.511	3.04×10^{-177}
	Daily alcohol consumption	0.005	0.001	0.076	1.11×10^{-5}
	Sex	-0.175	0.028	-0.108	3.62×10^{-10}
	SHIP cohort	-0.039	0.028	-0.022	0.164
	R2	*0.36367*			
Common risk factors including OCT1	Constant	-1.393	0.134		6.50×10^{-25}
	Age	0.009	0.001	0.155	2.00×10^{-20}
	BMI	0.093	0.003	0.51	3.80×10^{-177}
	Daily alcohol consumption	0.005	0.001	0.078	6.51×10^{-6}
	Sex	-0.175	0.028	-0.109	2.87×10^{-10}
	SHIP cohort	-0.04	0.028	-0.023	0.157
	Genetically predicted OCT1 activity score	0.07	0.026	0.044	5.82×10^{-3}
	ΔR2[+]	*0.00193*			

SE, standard error; [+] Change in explanatory power of the model after inclusion of OCT1 as risk factor

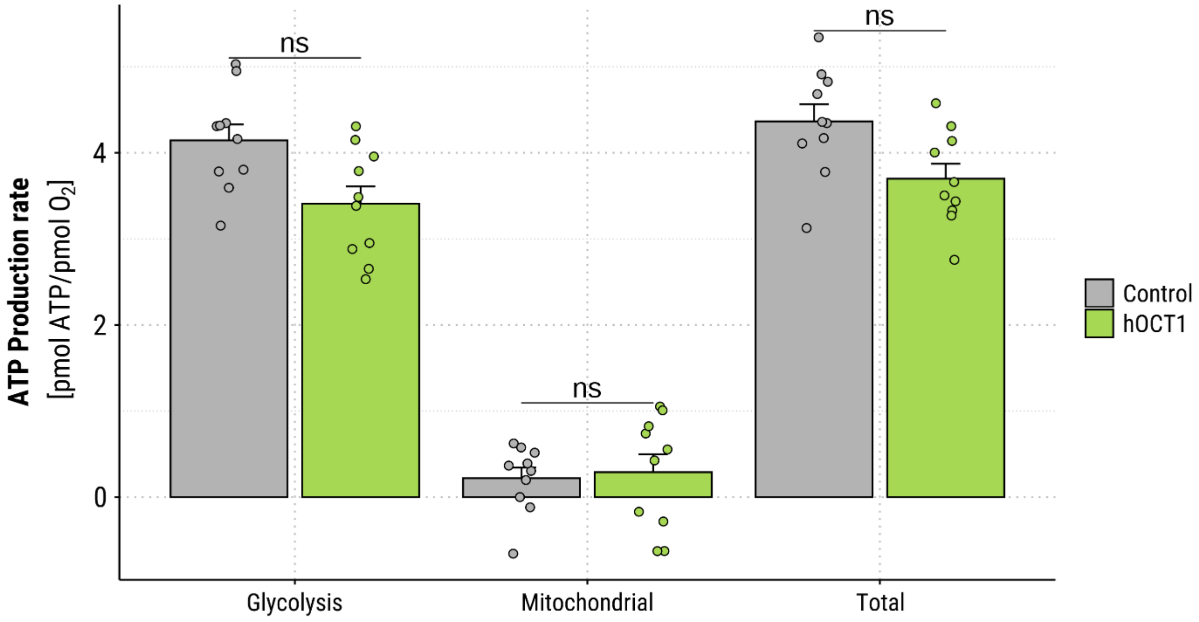

Supplementary Figure S1 - ATP production rates are not influenced by OCT1 overexpression under glucose as sole energy source. *A previously established Agilent Seahorse protocol was applied to HEK293 cells stably overexpressing human OCT1 (green bars) and control cells (pcDNA5, grey bars) to assess ATP production rates when glucose was the sole energy source. Cells were starved for 1 hour and consequently incubated with glucose as the sole energy source. Afterwards, inhibitors of the respiratory chain were injected to allow assessment of the ATP production rates. Data are presented as mean ± SEM from 3–6 independent experiments performed in duplicates. P values were calculated using unpaired Welch's t-tests with Bonferroni correction for multiple comparisons. ns $p \geq 0.05$*
